# Identification of Biomarkers for Resistance to *Fusarium oxysporum* f. sp. *cubense* Infection and *in Silico* Studies in *Musa paradisiaca* Cultivar Puttabale through Proteomic Approach

**DOI:** 10.3390/proteomes4010009

**Published:** 2016-02-24

**Authors:** Venkatesh Ramu, Krishna Venkatarangaiah, Pradeepa Krishnappa, Santosh Kumar Shimoga Rajanna, Nagaraja Deeplanaik, Anup Chandra Pal, Kukkundoor Ramachandra Kini

**Affiliations:** 1 Department of Post-Graduate Studies and Research in Biotechnology and Bioinformatics, Kuvempu University, Shankaraghatta 577 451, India; venka.biotech@gmail.com (V.R.); santosh09.kumarsr@gmail.com (S.K.S.R.); nagraja.dk@gmail.com (N.D.); 2 Department of Biotechnology Engineering, M S Ramaiah Institute of Technology, Bangalore 560 054, Karnataka, India; pradie.k@gmail.com; 3 Department of Studies in Biotechnology, University of Mysore, Manasagangotri, Mysore 570 006, India; chandrapalanup@gmail.com (A.C.P.); krk@appbot.uni-mysore.ac.in (K.R.K.)

**Keywords:** panama wilt, *Musa paradisiaca cv.* puttabale, PR proteins, two dimensional gel electrophoresis, protein-protein docking

## Abstract

Panama wilt caused by *Fusarium oxysporum* f. sp. *cubense* (Foc) is one of the major disease constraints of banana production. Previously, we reported the disease resistance *Musa paradisiaca cv.* puttabale clones developed from Ethylmethanesulfonate and Foc culture filtrate against Foc inoculation. Here, the same resistant clones and susceptible clones were used for the study of protein accumulation against Foc inoculation by two-dimensional gel electrophoresis (2-DE), their expression pattern and an *in silico* approach. The present investigation revealed mass-spectrometry identified 16 proteins that were over accumulated and 5 proteins that were under accumulated as compared to the control. The polyphosphoinositide binding protein ssh2p (PBPssh2p) and Indoleacetic acid-induced-like (IAA) protein showed significant up-regulation and down-regulation. The docking of the pathogenesis-related protein (PR) with the fungal protein endopolygalacturonase (PG) exemplify the three ionic interactions and seven hydrophobic residues that tends to good interaction at the active site of PG with free energy of assembly dissociation (1.5 kcal/mol). The protein-ligand docking of the Peptide methionine sulfoxide reductase chloroplastic-like protein (PMSRc) with the ligand β-1,3 glucan showed minimum binding energy (−6.48 kcal/mol) and docking energy (−8.2 kcal/mol) with an interaction of nine amino-acid residues. These explorations accelerate the research in designing the host pathogen interaction studies for the better management of diseases.

## 1. Introduction

*Musa paradisiaca* (L) *cv.* puttabale is an indigenous banana cultivar belongs to the AB genome [1,2] cultivated in the Malnad region of Karnataka, India. The fruits are valued for their delicious taste but are highly prone to *Fusarium oxysporum* f. sp. *cubense* (Foc) infection. Universally, farmers apply high dose of commercial fungicides and pesticides for the obliteration of this pathogen. However, the pathogen Foc has mutated, becoming increasingly resistant to fungicides and adopted to various environmental stresses thus posing an imminent threat for global banana production [3]. Conventional plants breeding techniques has been focused on disease resistant plants but are limited to several constraints such as polyploidy, heterozygosity, sterility, low fertility and limited genetic variability [4]. Alternatively, mutation induction, somaclonal variation and *in vitro* selection technologies have a prominent role in improving disease resistant traits [5]. Many investigators report the use of chemical mutagens such as ethyl-methane-sulfonate, diethyl sulfate, sodium azide [6,7] and the *Fusarium* culture filtrate or Fusaric acid [8,9] to improve *Fusarium* wilt resistant varieties of banana [10].

Genetic improvement by the insertion and expression of antifungal genes in the banana plant is an effective and sustainable management option to control *Fusarium* wilt. However, the insertion and expression of the anti-Foc gene in the banana plant has not been studied in detailed at the molecular level. Few studies have reported that over-expression of *Petunia* floral defensins, *PhDef1* and *PhDef2* (antimicrobial protein) in transgenic banana plants using *Agrobacterium*-mediated transformation. The significant expression of two defensin genes in elite banana *cv.* Rasthali have led to the development of resistance to *Foc* infections [11]. Similarly, Mahdavi *et al.* [12] demonstrated that the over-expression of the rice thaumatin-like protein gene in transgenic banana plants show enhanced resistance to *Foc* race 4. Apoptosis-like features in host plants are observed against necrotrophic pathogens, where the pathogen feeds off of the dead tissue thus increases its potential to grow rapidly. Only a few studies revealed the pathogen-induced defense genes in banana roots via the suppression subtractive hybridization method [13], and the expression patterns of genes involved in Foc4 pathogen-associated molecular pattern recognition in Cavendish banana roots [14]. Therefore, proteomic approaches have been used successfully to identify the proteins encoded by the genome and provide a direct insight into the signaling and metabolic processes coupled with the perturbation conditions. Banana proteomic research has made considerable progress in providing functional information about proteins accumulated in various developmental stages, tissues, cells in osmotic stresses and cold tolerance on banana growth and development [15,16]. Recently, advanced Mass spectrometery techniques, in conjunction with the *Musa* ssp. database has identified the 1131 unique proteins belonging to various biochemical pathways in banana fruit [17]. The sequencing of *Musa acuminata* ssp. *malaccensis* chloroplast also has been completed, and a reference sequence of the *Musa acuminata* nuclear genome has recently been made available in the public domain [18] which will lead to new insights in the proteomic analysis for genetic improvement of bananas [19,20,21]. Hence, it is imperative to understand the protein accumulation, expression patterns and molecular docking studies to target fungi in banana *cv.* puttabale against *Fusarium* infection. In a previous study, an attempt has been made to develop disease resistant cultivar of *Musa cv.* puttabale using EMS and Foc culture filtrate treatment [22]. A present investigation focuses on proteomic profiling and the validation of differentially accumulated proteins against Foc-inoculated *cv.* resistant and susceptible puttabale clones by using two-dimensional gel electrophoresis (2-DE). Homology modeling, molecular dynamic simulation, protein-protein and protein-ligand docking studies were studied against fungal targets.

## 2. Results

### 2.1. 2-DE 

**Figure 1:**
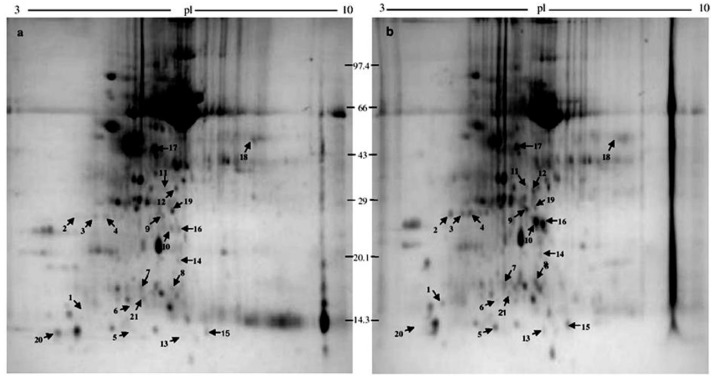
Two-dimensional gel electrophoresis (2-DE) profile of proteins. (**a**) Control banana *cv.* puttabale plant; (**b**) Foc infected banana *cv.* puttabale plant. **1** Pathogenesis-related protein; **2** Ring fyve phd zinc finger protein; **3** Dehydroascorbate reductase; **4** Salicylate o-methyltransferase-like; **5** Sucrose synthase; **6** Dynein heavy chain; **7** Pathogenesis-related protein; **8** Cadmium/zinc-transporting atpase; **9** 26s proteasome non-atpase regulatory subunit; **10** Ras-related protein rabb1c-like; **11** Alcohol dehydrogenase 1; **12** Polyphosphoinositide binding protein ssh2p; **13** Albumin-1 D; **14** Peptide methionine sulfoxide reductase chlo; **15** Disease resistance rpp13-like protein 1-like; **16** Subtilisin-like protease; **17** Protein odr-4; **18** Lrr repeats and ubiquitin-like domain-containing protein; **19** Auxin-responsive protein; **20** 60s acidic ribosomal protein p2a; **21** Leafy-like protein.

**Figure 2:**
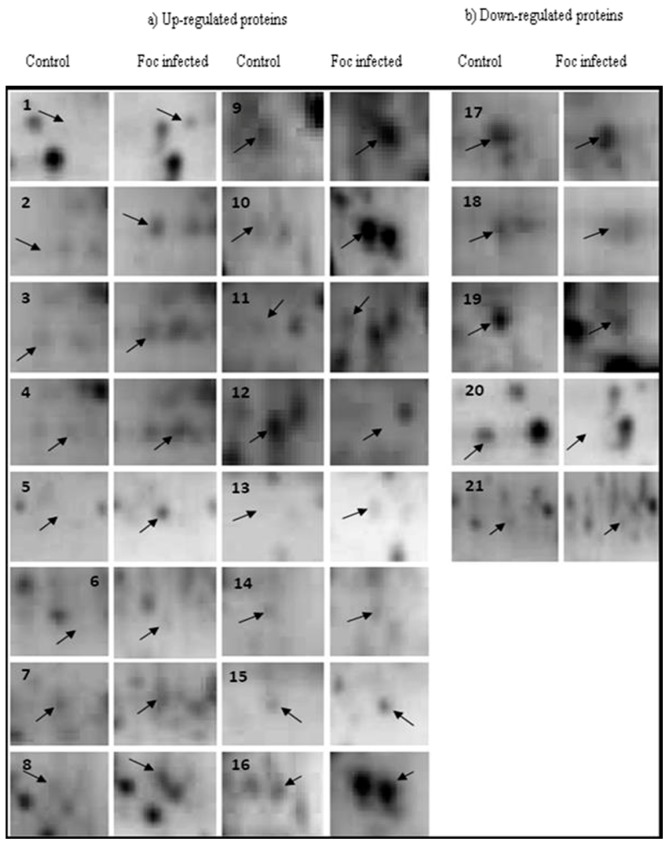
Resolved 2-DE images of differentially accumulated proteins. (**a**) Up-regulated protein spots upon infection of *Fusarium oxysporum* f. sp. *cubense* to banana *cv.* Puttabale; (**b**) Down-regulated protein upon infection of *Fusarium oxysporum* f. sp. *cubense* to banana *cv.* puttabale. **1** Pathogenesis-related protein; **2** Ring fyve phd zinc finger protein; **3** Dehydroascorbate reductase; **4** Salicylate o-methyltransferase-like; **5** Sucrose synthase; **6** Dynein heavy chain; **7** Pathogenesis-related protein; **8** Cadmium/zinc-transporting atpase; **9** 26s proteasome non-atpase regulatory subunit; **10** Ras-related protein rabb1c-like; **11** Alcohol dehydrogenase 1; **12** Polyphosphoinositide binding protein ssh2p; **13** Albumin-1 D; **14** Peptide methionine sulfoxide reductase chlo; **15** Disease resistance rpp13-like protein 1-like; **16** Subtilisin-like protease; **17** Protein odr-4; **18** Lrr repeats and ubiquitin-like domain-containing protein; **19** Auxin-responsive protein; **20** 60s acidic ribosomal protein p2a; **21** Leafy-like protein.

2-DEs of protein samples of both susceptible and resistant *Musa cv.* puttabale leaves after the 5th day of Foc inoculation showed the distribution of protein spots on the gel ranging from 10 kDa to 100 kDa, with isoelectric points (pI) ranging from 3 to 10. The colloidal coomassie stained gels were analyzed by PD-Quest software. It resolved an average of 300 ± 25 individual protein spots shown in the Figure 1a as Foc-inoculated susceptible plants and Figure 1b as Foc-inoculated resistant banana plants. The protein profile was highly reproducible among replicates from the same inoculation experiment with 180 ± 15 consistent spots. Upon normalization of protein spot images and manual verification, 70 differentially accumulated protein spots exhibited two-fold changes in the abundance value with a statistical significance of *p* < 0.05. Among these differentially accumulated proteins, 21 protein spots exhibited with high resolution on the gel of the Foc-inoculated plant sample. The PDquest software analysis revealed that the ratio of proteins accumulated at different ranges of relative abundance valuesare represented in Table S1. Among the 21 proteins spots exhibiting differential accumulation, only 15 protein spots were found to be truly significant as determined by Benjamini-Hochberg’s False discovery rate (FDR) correction method. Thus, the FDR proves to be an efficient statistical analysis method in revealing false positives in data, especially in the differential accumulation of proteins (Table S2). Among these 21 proteins, 16 proteins spots were over accumulated in resistant clones (Figure 2a; right side figures), and 5 protein spots were under-accumulated in susceptible clones (Figure 2b; left side figures). The 3D images of resolved differentially expressed proteins are demonstrated in Supplementary Figure S1.

### 2.2. Protein Identification

Based on the *m*/*z* values of a Matrix-assisted laser desorption/ionization Time of Flight (MALDI-TOF) analysis, 21 differentially accumulated proteins were searched against MASCOT search engine for their annotation (Table 1). Thus revealed that 18 proteins are specific to *Musa balbisiana* *cv.* “Pisung Klutuk Wulung” by sequence similarity in the Banana Genome Hub and the remaining three proteins in (National Center for Biotechnology) NCBI protein databases.

### 2.3. Gene Ontology

**Figure 3:**
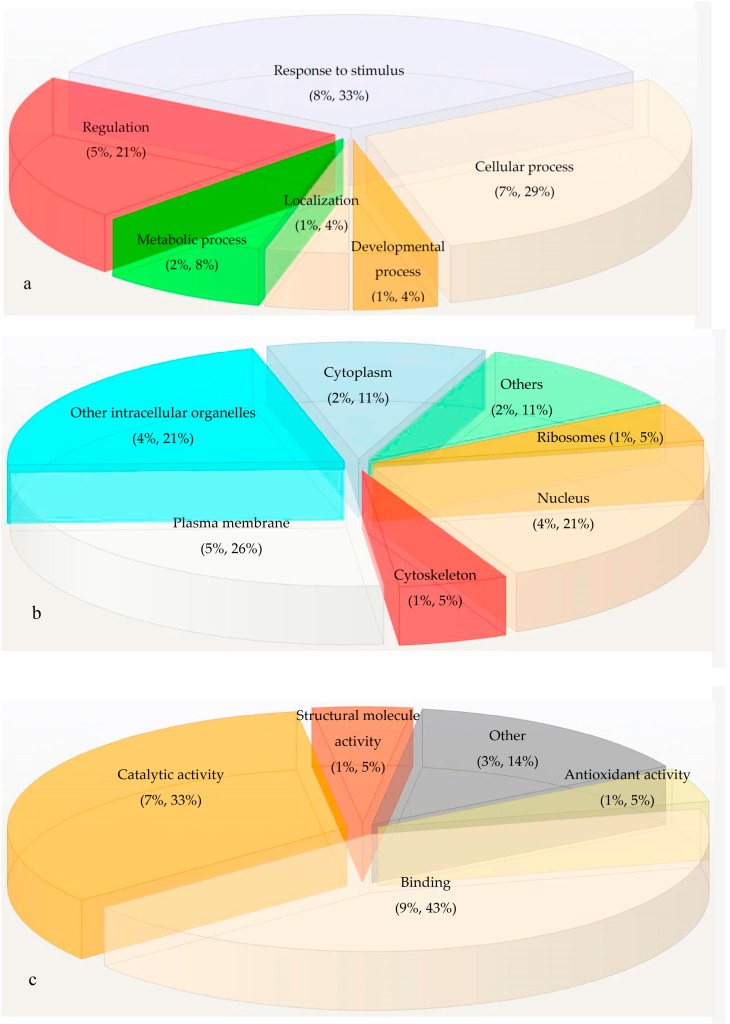
Gene ontology of differentially accumulated proteins using STRAP tool (**a**) Biological process; (**b**) Cellular process and (**c**) Molecular function.

*In silico* evaluation of 21 differentially accumulated proteins of Foc infected samples showed these proteins was ontogenically clustered into three groups such as biological process, cellular component and molecular function (Figure 3). Among these proteins, the nine proteins are concerned in binding purposes (43%), seven proteins exhibited catalytic activity (33%), one protein for antioxidant activity and one for related to structural molecule activity. This study revealed that a higher percentage of proteins perform the biological role in response to stimuli.

### 2.4. Quantitative Reverse Transcription PCR (qRT-PCR) Analysis

#### 2.4.1. RNA Extraction and Quantification

Total RNA extracted from infected and uninfected banana leaf material using an RNA mini kit showed an absorption ratio of 2.0 ± 0.1 at OD 260/280 nm and 1.5–2.0 at OD 260/230 nm measured with a Nanodrop ND-1000 spectrophotometer (Thermo Fisher Scientific, Wilmungton, DE, USA).

#### 2.4.2. Primer Design for qRT-PCR

The primers designed for *M. paradisiaca* BLAST hit sequences using Primer3plus online tool. The specificity of the PCR reactions of PR, PBPssh2p, PMSRc, IAA, disease resistant rpp13-like protein 1-like (DRrpp13) and reference gene tubulin was determined by a melting curve and an amplification plot to confirm the absence of primer-dimer formations. The resulting specific amplification peaks are represented in Figure S2a,b. The PCR products of differentially expressed genes were analyzed qualitatively on a 3.5% agarose gel which yielded a specific-sized amplicon and the profile of the gel is shown in Figure S3. The specific amplicon size of all the DEGs produced between 100 to 200 bp confirms the accuracy of the designed primers (Figure S3).

#### 2.4.3. Gene Expression

**Figure 4:**
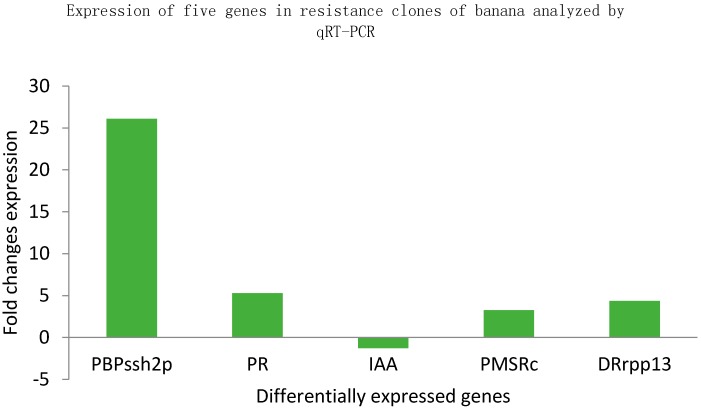
Expression of defense related proteins by qRT-PCR.

The qRT-PCR amplification of PR, DRrpp13, PMSRc, and PBPssh2p genes showed an increase in fold change expression. In contrast, the IAA gene showed a decreased fold change expression in resistant clones, as shown in Figure 4. The qRT-PCR experiment also highlighted that the gene PBPssh2p was significantly up-regulated with a 26.11 fold change and the gene IAA was down-regulated to a −1.27 fold change in *Fusarium* inoculated resistant clones as compared to susceptible clones.

### 2.5. Protein Homology Modeling

The differentially expressed genes namely, the pathogenesis-related protein (PR), peptide methionine sulfoxide reductase chloroplastic-like (PMSRc), polyphosphoinositide binding protein ssh2p (PBPssh2p) and indole acetic acid-induced-like protein (IAA) was subjected to a BLASTp query of the NCBI against the Protein Data Bank (PDB) database to know their structural similarity. It showed insignificant hits and query coverage for template-based homology modeling. The BLASTp analysis of PR amino acid sequences against the PDB database showed a similarity to the birch pollen allergen Bet V 1(PDB ID:1FM4_A) protein with an E-value: 9.31 × 10^-32^, bit-score: 114, aligned-length: 153, identity to query: 41% (Figure S4). Similarly, PMSRc showed a similarity with the peptide methionine sulfoxide reductase Msrb from the *Bacillus Subtilis* (PDB ID:2KZN_A) protein with an E-value: 1.04 × 10^-34^, bit-score: 124, aligned-length: 126, identity to query: 51% with query coverage 50%. (Figure S5), PBPssh2p showed a similarity with a functional phosphatidylinositol transfer from a pseudo-sec14 scaffold (PDB ID:3Q8G_A) protein with an E-value: 4.30 × 10^-19^, bit-score: 88.58, aligned-length: 198, identity to query: 35% with query coverage 37% (Figure S6) and IAA showed a similarity with the Aux/IAA transcriptional factors Ps-iaa4 of the *Pisum sativum* (PDB ID:2MIM_A) protein with an E-value: 1.57 × 10^-37^, bit-score: 132, aligned-length: 119, identity to query: 57% and query coverage 37% (Figure S7). The identity to query coverage of the four differentially accumulated proteins above is less than 60%. Therefore, all these accumulated proteins were subjected to *ab initio* homology modeling. 

### 2.6. Homology Modeling, Validation and Interaction Analysis

The Robetta server gave better structures of PR, PBPssh2p, PMSRc and IAA proteins. The validations of the modeled protein structure are as follows.

#### 2.6.1. PR Protein

The PR-modeled protein structure having 158 amino acid residues is shown in Figure S8a. The Ramachandran plot showed 92.6% residues in the most favored regions (Figure S8b). The proSA validation gave a surface energy z score of −5.92 and was found to be acceptable conformation with the desired range of native conformations (Figure S8c). The energy profile of proSA plot showed the energy per residue. Most residues in the model seemed to be having low energy scores (below the zero line) with the exception of minor peaks (Figure S8d). The MetaMQAPII of the PR protein has a Global Distance Test Total Score (GDT-TS) score of 83.228 and Root Mean Square Deviation (RMSD) values of 1.388, which results in a fairly good structure. The STRIDE server of the PR protein showed 9 coils, 66 β sheets, 40 α-helix and 43 β-turns residues.

#### 2.6.2. PMSRc Protein

The PMSRc protein model was built using 250 amino acid residues are shown in Figure S9a. The Ramachandran plot showed 89.0% residues in the most favored regions (Figure S9b). The proSA validation of the PMSRc modeled structure gave a surface energy z score of −7.08 and was found to be acceptable. The conformation and plot showed that the PMSRc model structure is within the desired range of native conformations (Figure S9c). The energy profile proSA model quality plot shows the energy per residue. The energy profile of most of the residues in the model seemed to have low energy scores (below the zero line) with the exception of a small variation in the peak observed above 0 (Figure S9d). The MetaMQAPII gave a GDT-TS score of 53.800 and RMSD values of 3.265, which is a good structure. The STRIDE server of the modeled PMSRc protein comprises 60 coils, 60 β sheets, 52 α-helix and 63 residues were in β-turns.

#### 2.6.3. PBPSSH2P Protein

The Robetta server gave a PBPssh2p protein model using 526 amino acid residues (Figure S10a). The Ramachandran plot of the modeled PBPssh2p protein showed 90.1% residues in the most favored regions (Figure S10b). The proSA validation of the PBPssh2p modeled structure gave a surface energy z score of −8.9 and was found to be conformational acceptable. The plot showed that the PBPssh2p model structure is within the desired range of native conformations (Figure S10c). The energy profile of most residues in the model seemed to have low energy scores (below the zero line) with the exception of small variations in the c-terminal region (Figure S10d). The MetaMQAPII showed a GDT-TS score of 48.241 and RMSD values of 3.914. The STRIDE server analysis of the PBPssh2p protein contains 98 coils, 40 β sheets, 219 α-helices and 130 β-turn residues.

#### 2.6.4. IAA Protein

The Robetta server gave an IAA protein model using 319 amino acid residues (Figure S11a). The Ramachandran plot of the modeled IAA-induced protein showed 90.5% residues in most favoured regions (Figure S11b). The proSA validation of the IAA modeled structure gave a surface energy z score of −6.46 and was found to be conformationally acceptable. The plot showed that the IAA model structure is within the desired range of native conformations (Figure S11c). Most residues in the model seem to have low energy scores (below the zero line) with the exception of one minor peak observed (Figure S11d). The MetaMQAPII gave a GDT-TS score of 46.082 and RMSD values of 4.082. The STRIDE server analysis of the IAA protein revealed 76 coils, 36 β sheets, 120 α-helices and 69 residues in β-turns.

### 2.7. Active Pocket Prediction

The detection of the major binding pocket of modeled proteins was analyzed using a Computed Atlas of the Surface Topography of proteins (CASTp) revealed that the PR has an active pocket of area about 1141.8 Å^2^ and a volume of about 1311.8 Å^3^. The PMSRc protein consists of 70 amino acid residues within an area about 2039.5 Å^2^ and a volume of about 3128.3 Å^3^. The PBPssh2p protein has a major active pocket with an area of 6837.6 Å^2^ and a volume of 19409 Å^3^. The IAA protein showed major active pocket which covered an area of 3514.6 Å^2^ and a volume of 5846.8 Å^3^. Simultaneously, the major fungal protein endopolygalacturonase (PG) protein subjected to a CASTp analysis showed a major active site with 28 amino acid residues in an area of 458.9 Å^2^ and a volume of 788.3 Å^3^.

### 2.8. Molecular Dynamic Simulation (MDS)

The structural stability of proteins was determined by several parameters such as RMSD, Rg (radius of gyration), RMSF (root mean square deviation) and potential energy during the course of MDS. In the present study, the MDS of the PR protein over the time of simulation showed a plot of RMSD which gradually increased to 5000 ps and there was a slight fluctuation in structure simulation (Figure S12a). The Rg was stable during the simulation of 5000 ps (Figure S12b). Beyond this, no conformational changes were observed and structure became more complex. The RMSF values of the PR protein demonstrate the C-α atoms plotted against the residue number over the time period 7000–12,000 ps as shown in Figure S12c. From the graph it was observed that the first few residues of the proteins are fluctuated and a major fluctuation was noticed at the residue stretches between 75–100, 150–200, 250–300 and 350–400 ps during the course of simulation. Apart from the first few residues, a profound fluctuation was also observed between the residue of 150–200 ps. The RMSD plot of PMSRc showed that the structure of the protein gradually increased to 5000 ps and a stable structure was formed within 0.4–0.5 nm (Figure S13a). The Rg was decreased during the 1000 to 5000 ps time frame within 2.0 nm (Figure S13b). The RMSF of the C-α atoms was plotted against the residue number over the time period 100–800 ps within 0.1–0.4 nm (Figure S13c). The PBPssh2p protein showed an RMSD plot at 5000 ps within 0.8 nm (Figure S14a) and the time of Rg varied at 1000 to 5000 ps within 2.75 nm of simulation (Figure S14b). The RMSF exhibited the C-α atoms plotted against the residue number over the time period 1500 ps within 0.2–0.6 nm (Figure S14c). Similarly, the RMSD plot of the protein IAA increased to 5000 ps within 0.5 nm (Figure S15a) and there was no variation during the Rg time evolution at 3000 to 5000 ps (Figure S15b). The time period for the RMSF plot of this protein was 100–1000 ps (Figure S15c). The total potential energy of the modeled proteins viz., PR (Figure S16a), PMSRc (Figure S16b), PBPssh2p (Figure S16c) and IAA (Figure S16d) proteins did not exceed −9.6 × 10^5^ kJ/mol, indicating that these proteins were thermodynamically stable in itheir structures. Therefore, in further investigations these defense related differentially accumulated modeled proteins were subjected to protein–protein docking studies with the fungal pathogenic protein endopolygalacturonase. 

### 2.9. Docking Studies

#### 2.9.1. Protein-Protein Docking

The docking studies of the fungal protein endopolygalacturonase (PG) with differentially accumulated proteins namely, PR, PBPssh2p, PMSRc, and IAA using a Global Range Molecular Matching (GRAMM-X v.1.2.0, Vakser Lab, Center for Computational Biology, The University of Kansas, 2030 Becker Drive, Lawrence, KS, USA) server showed the following integration of proteins. The ten models of the PR protein complex with PG obtained from the GRAMM-X server were studied for their interactions. Among them, Model-III showed better protein interactions. The Figure S17a,b showed the cartoon view and molecular surface view of the PG/PR complex. The PR protein binds to the active pocket (highlighted in yellow color) of the PG protein. The docking of PG/PR proteins was evidenced by three ionic interactions, seven hydrophobic interaction, electrostatic energy (25.2598 kJ/mol), Van der Waals energy (372.17 kJ/mol), total stabilizing energy (391.71 kJ/mol), total accessible surface area (19,186.6 Å^2^), buried area (2171.4 Å^2^), solvation free energy gain upon complex formation (−11.0 kcal/mol), free energy of assembly dissociation (1.5 kcal/mol), P-value for interface specificity (0.232), one salt bridge and six hydrogen bonds are formed across the interface. The interaction values of all the ten models of the PR protein complex with PG protein are showed in Table S3. 

The docking of the PMSRc protein with the PG molecule showed confirmatory interactions in Model-IV. Figure S18a,b show the cartoon view and molecular surface view of the PMSRc/PR complex. The result revealed that the one ionic, six hydrophobic interaction, electrostatic energy (23.9878 kJ/mol), Van der Waals energy (−91.8147 kJ/mol), total Stabilizing Energy (−62.7369 kJ/mol), total accessible surface area (25,613.5 Å^2^), buried area (2004.5 Å^2^), solvation free energy gain upon complex formation (−8.8 kcal/mol), free energy of assembly dissociation (1.5 kcal/mol), *p*-value for interface specificity (0.109) and thirteen hydrogen bonds formed across the interface. Here, no salt bridge was formed between protein–protein interactions (Table S4).

The PBPssh2p protein docked with the PG molecule showed a better interaction structure in Model-II. Figure S19a,b that the cartoon view and molecular surface view of the PBPssh2p/PR complex. The interaction resulted in one ionic interactions, three hydrophobic interaction, electrostatic energy (32.789 kJ/mol), Van der Waals energy (432.02 kJ/mol), total stabilizing energy (464.209 kJ/mol), total accessible surface area (42,795.4 Å^2^), buried area (2356.4 Å^2^), solvation free energy gain upon complex formation (−15.2 kcal/mol), free energy of assembly dissociation (4.0 kcal/mol), P-value for interface specificity (0.053), six hydrogen bonds and no salt bridge, across the interface (Table S5).

Similarly, the docking of the IAA induced protein with the PG molecule showed better interactions in Model-I. Figure S20a,b shows the cartoon view and the molecular surface view of the IAA/PR complex. with one ionic and five hydrophobic interactions, electrostatic energy of −13.4389 kJ/mol, Van der Waals energy of 698.76 kJ/mol, total stabilizing energy of 683.351 kJ/mol, total accessible surface area of 30,208.4 Å^2^, buried area of 2943.3 Å^2^, solvation free energy gain upon complex formation of −16.9 kcal/mol, free energy of assembly dissociation of 7.6 kcal/mol and a *p*-value for interface specificity of 0.136. One salt bridge and nine hydrogen bonds are formed across the interface as mentioned in the Table S6.

#### 2.9.2. Protein-Ligand Docking

A molecular docking of the modeled PR, PBPssh2p, PMSRc and IAA proteins was performed with two fungal constituents namely, β 1, 3- glucan and chitin using the AUTODOCK program. All amide bonds were set rigidly and all ligand bonds were allowed to move freely based on the docking of several energies, *i.e.*, free energy binding energy (ΔGb), inhibition constant, intermolecular energy, torsional energy, RMS and internal energy.

##### Docking of Modeled Protein with β 1, 3-Glucan

The highest binding energy correlates with the highest binding efficiency in protein and drug interactions. In PR protein docking, the following amino acid residues play an active role in the interaction with β 1, 3 glucan viz., SER3:O-GLC401:H6, ASP127:OD2-BGC402:H2, THR132:HG1-BGC402:O2, SER5:HN-BGC402:O6, SER5:O-BGC403:H2, SER5:O-BGC402:H6, THR:O-BGC403: H3 (Figure S21a–c). Of the 10 docked confirmations, the 9^th^ one is the best confirmation because β 1, 3 glucan binds with seven hydrogen bonds and has the highest binding energy (−7.55 kcal/mol) and an inhibition constant of 2.94 × 10^-6^ as shown in Table S7. The PMSRc proteins interacted with the β-1, 3 glucan compound by the involvement of nine amino acid residues namely, GLN59:OE1-BGC403:H6, ASP100:OD2-BGC402:H6, LYS246:HZ3-BGC402:O3, ASN114:HD22-BGC402:O4, LEU247:O-GLC401:H2, ASN114:HD21-BGC403:O2, GLN68:OE1-GLC401:H4, GLN68:HE21-GLC401:O3 and ARG134:O-BGC403:H3 (Figure S22a–c). These amino acids play a active role in binding the β-1, 3 glucan compound. Out of 10-docked complexes, the ninth one is the best conformation because β 1, 3 glucan binds with highest binding energy (−6.48 kcal/mol) and an inhibition constant of 2.94 x 10^-006^ (Table S8). The docking of PBPssh2p proteins with β 1, 3 glucan resulted in the involvement of five amino acids in the interaction viz., VAL97:O-BGC402:H3, LYS137:O-GLC401:H6, VAL97:O-BGC402:H2, GLN89:HE21-BGC402:O6 and GLU252:OE2-BGC403:H6 (Figure S23a–c). Of 10 docking complexes, the fifth conformation showed the best result because β 1, 3 glucan binds with the highest binding energy (−8.03 kcal/mol) with an inhibition constant of 1.3 × 10^-6^ (Table S9). Similarly, binding energy of IAA protein with the β-1, 3 glucan was −8.92 kcal/mol with inhibition constant of 2.91 × 10^-007^ (Table S10). Of the 10 docked conformations, the first is the best interaction because eight amino acid residues play an active role in this conformation viz., ALA102:O-BGC402:H2, GLY78:HN-BGC402:O2, SER76:O-BGC402:H3, ASP80:HN-GLC401:O1, ASP219:OD2-BGC402:H6, TRP105:HE1-GLC401:O4, PRO84:O-GLC401:H6 and SER76:O-BGC403:H6 (Figure S24a–c).

##### Docking of Modeled Proteins with the Fungal Chitin

The docking result of the chitin molecule with four proteins namely, PR, PBPssh2p, IAA, PMSRc is as follows. Each modeled protein was separately docked with the chitin oligomer and the best-docked complex was analyzed. The PR protein interacts with the chitin substrate by two amino acids namely, SER5:HG-NAG703:O3, LYS114:HZ3-NAG702:O7 (Figure S25a–c). These amino acid residues were involved in this conformation with binding energy (−0.93 kcal/mol) and an inhibition constant of 0.21 to the chitin molecule (Table S11). The docking of the PMSRc protein with the chitin molecule showed the involvement of Four amino acids in the interaction namely, GLY66:O-NAG700:O1, GLY66:O-NAG700:O5, GLN68:OE1-NAG700:O3 and GLN102:HE21-NAG700:O1. (Figure S26a–c). It resulted in the highest binding energy (−1.92 kcal/mol) and an inhibition constant of 0.04 (Table S12). The PBPssh2p proteins interacted with the chitin substrate by the involvement of four amino acid residues namely, GLN5:O-NAG700:O4, ARG289:HH12-NAG700:O6, GLN:O-NAG701:O5 and GLY144:HN-NAG701:O6 (Figure S27a–c) and these amino acids played a active role in binding the chitin substrate. Out of 10 docked complexes, the ninth is the best conformation because chitin binds with the highest binding energy (−2.29 kcal/mol) and an inhibition constant of 0.02 (Table S13). Similarly, the IAA protein docked with the chitin molecule showed two amino acids present at the site of interaction. The residues ASN183:O-NAG700:O5 and SER261:HG-NAG702:O7. These amino acids play an active role in this conformation (Figure S28a–c) with a binding energy (−3.82 kcal/mol) and an inhibition constant of 0.0 (Table S14).

## 3. Discussion

*M. paradisiaca cv.* puttabale is an indigenous banana of Karnataka state, India. Needs to be reworded such as high frequency regeneration of plantlets through leaf calli [23] and immature male flower calli [24]. Further, the screening of *Fusarium* wilt disease resistant micropropagated clones through the assay of biomarkers, such as phenylalanine ammonialyase, peroxidase, polyphenol oxidase, catalase, chitinase and β-1-3 glucanase [22]. In the present study, the resistant and susceptible regenerants were acclimatized in a greenhouse for a period of two months and used for fungal pathogen inoculation by the root dipping method. The pathogen inoculated susceptible plants showed symptoms of *Fusarium* wilt, an initial appearance of slight vein clearing on the outer portion of the younger leaves, then epinasty (downward drooping) followed by stunting, chlorosis, yellowing of the lower leaves, wilting of leaves and young stems. Later, there was defoliation and necrosis of remaining leaves and finally, the whole canopy died. But the resistant clones did not show any sign of panama wilt. After the 5th day of inoculation, the third leaf of both resistant and susceptible clones was detached and immediately frozen in liquid Nitrogen for protein profiling. An average 300 ± 25 individual protein spots were resolved well with high poise. Lu *et al.* [21] reported the proteomic profiling of a susceptible variety (Williams) and a resistant variety (GCTCV-119) respond to TR 4 roots upon Foc inoculation. This yields 800 proteins spots which were resolved well and detected on each 2-D gel using PD-Quest v.8.01 software. Similarly, a proteomic profile analysis of three different banana cultivars namely, susceptible “Brazil”, moderately resistant “Nongke No.1” and highly resistant “Yueyoukang I” upon *Fusarium oxysporum* f. sp. *cubense* tropical race 4 inoculation was reported [20]. The results revealed that β-1, 3-glucanase and chitinase were found only in pathogen-challenged plants of the resistant variety compared with the susceptible plants. The present study also aims at protein profiling and *in silico* studies of pathogen related proteins in resistance and susceptible samples.

Pathogenesis-related proteins (PR proteins) are a group of diverse proteins whose accumulation is activated by a pathogen encounter. They play a great role in natural defense by changes in ion fluxes across the plant cell membrane, generation of reactive oxygen species (ROS), changes in the phosphorylation state of regulatory proteins and transcriptional activation of plant defenses at the site of infection, local accumulation of phytoalexins and cell wall rigidification due to callose, lignin and suberin deposition. In the present investigation, a qRT-PCR analysis revealed that the PR, PMSRc, PBPssh2p, DRrpp13 and IAA genes were differentially expressed. These proteins may accumulate locally at the site of infection, and systematically in the whole plant as part of systemic-acquired resistance against Foc infection. In response to an incompatible pathogen attack, plants produces more ROS and coincidently reduce ROS-scavenging activities, which results in the accumulation of ROS and the activation of the hypersensitive response (HR) [25]. The most of the methionine sulfoxide reductases B (MsrB) proteins show enhanced accumulation in response to plant dehydration and oxidative stress [26]. It is also reported that MsrB, which is an important ROS-modulating enzyme might play a key factor in plant defense by regulating the oxidation/reduction state of methionine (Met) and the oxidative state of the cells. ROS readily oxidizes Met residues in proteins/peptides to form Met-Rsulfoxide or Met-S-sulfoxide, resulting in the inactivation or malfunction of the proteins. Methionine sulfoxide reductases catalyzes the reduction of methionine sulfoxide (MetSO) to methionine in proteins and plays a protective role against oxidative stress by restoring activity to proteins that have been inactivated by methionine oxidation [27,28,29,30]. Swarupa *et al.* [31] reported current research on oxidative burst and antioxidant enzymes, *i.e.*, ROS plays an important signaling molecule in the banana defense response against *Fusarium oxysporum* f. sp. *cubense*. In the present investigations, an anti-oxidative stress protein PMSRc was up-regulated after Foc inoculation with 3.27 fold changes. The protein PBPssh2p was involved in lipid metabolism and important cellular activities associated with a trans-Golgi network, membrane trafficking and membrane biogenesis [32]. Here, the up-regulation of the PBPssh2p protein occurs with a 26.11 folds change. In contrast to up-regulated proteins, a down-regulation of the IAA-induced protein was noticed after Foc-inoculated clones. The over-accumulation of the IAA-induced protein could act as a defense factor of the host plant by phenolic infusion, glucanase synthesis, gel formation, phytoalexin synthesis and tylose formation [33]. Fernandez-Falcon *et al.* [34] also reported that the exogenous applications of the IAA could significantly increase the resistance against *Fusarium* wilt in banana. In the present study, the IAA-induced protein was down-regulated with −1.27 folds indicating the repression of IAA responsive gene in clones against *Fusarium* inoculation. The MD simulation of proteins was evaluated by several parameters such as RMSD, RMSF, Rg for the deviation of the structure, and compactness and flexibility of each residue over the period of the simulation. Subarna *et al.* [35] reported that the molecular dynamics simulation studies of PR-1 protein from *Solanum tuberosum* revealed the flexible nature of the residues (115–120) of the fourth α-helix which provided a clue in the identification of this region as functionally important. Similarly, the PMSRc and PBPssh2p proteins retained an overall backbone structure and its non-covalent interaction and showed fluctuations in the structural correspondence of the residues to facilitate the access of the active site. Ferrari *et al.* [36] reported that the plant protein endopolygalacturonase inhibiting protein (PGIP) interaction with the fungal protein endopolygalacturonase (PG) controls the destructive potential of the PG. The partial degradation of homogalacturonan from PG elicits oligogalacturonides. These oligogalacturonides are capable of producing defense responses, including the accumulation of Reactive oxygen species (ROS) and pathogenesis-related proteins. They also protect the plants against pathogen infections and also limit pathogen penetration and tissue colonization by inhibiting PG activity [37]. Hence, PGIP seems to exert a dual role during fungal attack. Maulik *et al.* [38] reported that the homology modeled endopolygalacturonase inhibiting protein (PvPGIP1), PvPGIP2 of *Phaseolus vulgaris* and the Glycine max endopolygalacturonase inhibiting protein (GmPGIP3) were docked with PG of *Fusarium moniliforme* (FmPG). The result revealed that the PvPGIP1 was incapable of inhibiting the FmPG. The model FmPG docked to PvPGIP1 at the face is similar to the one in PvPGIP2 of the PvPGIP2-FmPG complex. However, GmPGIP3 possesses an inhibiting activity towards the PG of *F. moniliforme* based on electrostatic interaction and hydrogen bonds. In the present study, the PR protein docked to the PG molecule with high affinity. This interaction is evidenced by the solvation free energy gain upon complex formation (−11.0 kcal/mol), the free energy of assembly dissociation (1.5 kcal/mol) and the formation of six hydrogen bonds are formed. Hence, the PR protein could be consider a potent antiPG protein.

Pathogenesis related proteins are able to hydrolyze various components of the fungal cell wall (glucans and chitin), for example fungal cell wall degrading enzymes (e.g., β-1,3-glucanases [PR-2] and chitinases [PR-3/8/11]) and serine and cysteine proteases (e.g., P69 and Rcr3), respectively [39,40]. Yadav *et al.* [41] reported the recognition of fungal β-glucans by dectin-1 that leads to multiple cellular responses, some of which are host protective such as fungal uptake and killing and produce inflammatory cytokines and chemokines. Interestingly, dectin-1 also induces the production of IL-10, an anti-inflammatory cytokine whose role during fungal infection which leads to the activation of leukocytes may be important for limiting host injury during severe inflammation. Here, the PMSRc protein showed the best binding conformation to β-1, 3 glucan with nine hydrogen bonds and had the highest binding energy (−6.48 kcal/mol). Zhu *et al.* [42] reported that the binding of the PR-4 members with the chitin in the developing fungal cell wall results in fungal growth retardation. The molecular docking of the CDA1 protein from *Bacillus thuringiensis* substantiates the chitin binding affinity with −6.5 kcal/mol [43]. The negative and low value of binding energy as well as maximum hydrogen bonding showed strong and most favorable binding between protein and ligand molecules [41]. In the present study, the PMSRc and IAA protein showed good binding affinity of −3.82 kcal/mol and −1.92 kcal/mol respectively. In the present study, a protein profiling of *M. paradisiaca cv.* puttabale against Foc infection was carried out. The results revealed that a few proteins are involved in biological procesess such as regulation, cellular procesess, response to stimuli, antioxidant activities *etc.* The qRT-PCR analysis validated the imperative genes namely PR, DRrpp13, PMSRc, and PBPssh2p showed up-regulation whereas, IAA showed down-regulation in leaf sample. The protein profiling, signaling network, protein-protein interaction in banana plants have gained great importance in analyzing in the disease-resistant mechanism against the panama wilt of *M. paradisiaca cv.* puttabale. The three-dimensional structure prediction of the most plausible candidate protein proposed that it may be used further to understand the potential mechanism of disease and the role of these proteins in controlling diseases.

## 4. Materials and Methods

### 4.1. Inoculum Preparation and Sampling

Foc was isolated from infected *M. paradisiaca cv.* puttabale grown in a farmyard of Shimoga district, Karnataka, India. The isolated Foc pathogen was identified and authenticated by Dr. Chowdappa P, Principal Scientist, Division of plant pathology, Indian Institute of Horticulture Research, Bangalore, Karnataka state, India and confirmed by an Internal Transcribed Spacer (ITS) sequence analysis [44]. The 10^6^ spores/mL suspension were used as an inoculum. The Foc were inoculated to the roots of two month old *in vitro* regenerants resistant and susceptible clines for 30 min and then replanted them in a pot.

### 4.2. Protein Extraction and 2-D Gel Electrophoresis

The third leaves of the 5th day of Foc which inoculated two month old hardened resistant and susceptible regenerants were sampled for protein extraction as per the protocol of the Phenol extraction methanol/ammonium acetate precipitation method [24] and dissolved in a rehydration buffer (7 M urea, 2 M thiourea, 4% CHAPS, 1% IPG buffer, 1% DTT). The quantification of protein was done by the Bradford method using a NanoDrop ND-1000 spectrophotometer (Thermo Fisher Scientific, Wilmungton, DE, USA) and the protein samples were stored at –20 °C for future use. The rehydration buffer containing protein samples (250 µg in 180 µL) was diluted to a 0.002% bromophenol blue solution, mixed and loaded into pre-hydrated 11 cm, pH 3–10 IPG strips (Cat. No: 163-2014, BioRad, Hercules, CA, USA). For isoelectric focusing, immobilized polyacrylamide gel (IPG) strips were initially run with 250 V for 20 min (linear), then 8000 V for 150 min (linear) and finally run with 25,000 V for 150 min (rapid). Prior to the second dimension, the individual strips were equilibrated with an equilibration buffer [45]. Second dimension electrophoresis were carried out with a lab cast 1.5 mm thickness of Sodium do-decyl sulfate polyacrylamide gel (12.5%) and run for 90 min at a constant 200 V. Standard protein markers were obtained from Genei, Bangalore, India (Cat. No: 105695). The gels were stained with a colloidal coomassie brilliant blue stain (10% ammonium sulfate, 1% phosphoric acid, 5% coomassie brilliant blue (CBB) G-250) for 24 h and destained with double distilled milli-Q water. 

The gel images were processed using Gel Doc-XR (BioRad) and analyzed with PD-Quest software (BioRad) using a 10-fold over the background as a minimum criterion for spot presence/absence. The normalization of spot intensities between the control and pathogen inoculated samples were carried out to detect differentially accumulated proteins by the densitometric method. Normalized spot intensities (individual spot intensity/normalization factor, calculated for each gel based on total density in gel image) were determined for each spot accumulated in parts per million (PPM). A statistical analysis of normalized spots was performed using Student’s *t*-test (*p* < 0.05). The significance level was further corrected by the Benjamini-Hochberg false discovery rate procedure [46]. The spots showing a 2-fold protein accumulation increase or decrease were used for a mass spectrometric analysis.

### 4.3. MALDI-TOF-MS Analysis

Protein spots were excised manually from gels and used for trypsin digestion as per the method [47]. One μL of a trypsin-digested sample was deposited onto the MALDI target and analyzed with a MALDI-TOF-TOF (Ultra Flex TOF-TOF, Bruker Daltonics, Bremen, Germany) instrument equipped with a N_2_ laser, and analyzed in reflectron mode using a time delay of 90 ns and an accelerating voltage of 25 kV in positive ion mode. The study was carried out with the help of the proteomic facility unit, Indian institute of Science, Bangalore. The background ions from trypsin autolysis and keratin contamination were removed from mass lists. A protein identification was performed by searching with Viridiplantae in the latest version of the NCBI database, against *Musa balbisiana* cultivar “Pisung Klutuk Wulung” in the Banana Genome Hub [48] and in Swiss-Prot using the Mascot search engine [49]. The parameters of monoisotopic mass accuracy, maximum 1.2 Da; maximum missed cleavages, 3; allowed variable modifications, oxidation (Met) and fixed modification. Carbamidomethyl (C) was applied.

### 4.4. Gene Annotation

Gene ontology analyses of 21 differentially expressed proteins were performed by using STRAP (Software Tool for Researching Annotation of Proteins) tool [50]. The Uniprot ID of 21 differentially expressed proteins were retrieved from UniProt identifiers (The European Bioinformatics Institute, Wellcome Trust Genome Campus, Hinxton, Cambridge, UK) and upload to STRAP tool to generates protein annotation tables and a variety of GO charts of differential analysis of proteomics data.

### 4.5. qRT-PCR Analysis

The total RNA was extracted from leaf samples of both resistant and susceptible banana clones using a plant total RNA mini kit (cat#YRP50, Real Biotech Corporation, Taiwan). The quality and quantity of RNA was measured using a NanoDrop ND-1000 spectrophotometer. Then cDNA was synthesized from isolated RNA using a First Strand cDNA synthesis kit (cat#K1611, Fermentas, 7520 Connelley Dr. Suite A Hanover, MD, USA) with Oligo[dT] primers. About 2 μg of total RNA in a single 20 μL reaction was quantitatively converted to single-stranded cDNA. The primer pairs were designed to gene sequences of *Musa balbisiana* cultivar “Pisung Klutuk Wulung” available in the Banana Genome Hub. The gene sequences using Primer3plus software [51] and cross validated with a NetPrimer program (PREMIER Biosoft International, Palo Alto, CA ) (Table S15). The optimum parameters were set as a melting temperature (Tm) of 60 °C, a primer size of 20–24 nucleotides, a GC content of 45%–55% and a product size of 100–150 base pairs. The specificity of primer pairs was confirmed by using a BLAST analysis in NCBI against *Musa balbisiana* cultivar “Pisung Klutuk Wulung” sequences. A PCR mixture of 10 μL containing 5 μL of VeriQuest SYBR Green qPCR Master Mix (2X) (Product number 75600, Affymetrix, Inc, Cleveland, OH, USA), 1 μL of diluted cDNA, 500 nM of each gene-specific primer and an appropriate amount of sterile ddH20 was mixed freshly for the qRT-PCR experiment. The experiment was performed on the Applied Biosystems 7500 Real-Time PCR System (Applied Biosystems, Grand Island, NY, USA) to monitor DNA synthesis. The qRT-PCR program includes, initial denaturation at 95 °C for 10 min followed by 40 cycles of denaturation at 95 °C for 15 s, and annealing and extension at 60 °C for 60 s. A melting curve analysis was determined for all the reactions and expression level was calculated by the 2^−ΔΔCt^ method [52]. The gene tubulin was used as a reference for normalizing the expression data to measure the response of predicted pathogen responsive genes.

### 4.6. Sequence Alignment and Homology Modeling

The differentially accumulated proteins namely, PR, PBPssh2p, PMSRc and IAA were searched against the PDB by BLASTP for the presence of similar three-dimensional protein structures. The structure predictions were made using the *de novo* Rosetta method by using Robetta server [53] which provides both *ab initio* and comparative models of protein domains. Domains without a detectable protein homology were modeled with the Rosetta *de novo* protocol. The structure analysis and verification server v4 (SAVES; University of California, Los Angeles, CA, USA) was used for assessing the modeled protein structures. The energy profiles of the modeled proteins were evaluated by using a ProSA server [54].

The quality of the computational models of protein structure was assessed by a MetaMQAPII server [55]. The absolute deviation of the C-α atom (backbone atoms) in each residue expressed as RMSD and GDT-TS [56] of the modeled proteins was calculated using a MetaMQAPII server. The STRIDE server provides an interactive interface to secondary structure proteins [57]. The active pocket of the proteins was identified using CAST_P_ with the radius set to 1.4 Å [58]. 

### 4.7. Molecular Dynamic Simulation

The constructed refined model of the PR, PBPssh2p, PMSRc and IAA proteins were used for the MDS studies. The simulation was performed using the Groningen Machine for the Chemical Simulation (GROMACS) program [59] running on an ubuntu Linux platform, version 12.04, in an HP Z600 workstation having a clock speed 2.13 GHz Quadra core processor enabling parallel computation. The starting structure was solvated in a cubic box with a dimension of 10 Å consisting of simple point-charge (SPC) water molecules added inside a specified cubic periodic boundary of 0.75 nm distance from the center using editconf and genbox commands. The protein simulation was carried at the constant temperature of 300 K, by coupling each component separately to a temperature bath using the Berendsen coupling method [60]. Before running the simulation, the solvent was relaxed by energy minimization where 1000 steps of steepest descent (converging in 128 steps) were used followed by 1000 steps of a conjugant gradient process (converging in 10 steps). This was followed by 3.0 ns of simulation imposing positional restraints on the non-H atoms. The positional restraints were released and the 10 ns production run was analyzed. The trajectory files obtained during the simulation were used to calculate RMSD, root mean square fluctuation (RMSF), radius of gyration (Rg) and potential energy in the GROMACS package.

### 4.8. Protein-Protein Docking

A GRAMM-X web server [61] was utilized for protein-protein docking of PR, PBPssh2p, PMSRc and IAA proteins with fungal protein endopolygalacturonase (PG; PDB ID: 1HG8) in order to unveil the mode of interaction within and across each pair. The output of GRAMM-X in a PDB file containing the structures of 10 models was ranked as the most probable prediction candidate and a post-docking analysis was carried out using Pymol software v1.3 Schrodinger, LLC, NY, USA.

### 4.9. Determination of Protein-Protein Interactions

A protein Interaction Calculator was used to determine the ionic and hydrophobic interaction between the protein complexes [62]. A Coil check online tool was used to determine the electrostatic energy, Van der waals energy and total stabilizing energy between the complexes [63]. A proteins, Interfaces, Structures and Assemblies (PDBePISA) interactive tool was used to find out the total accessible surface area, buried area, solvation free energy gain upon complex formation, free energy of assembly dissociation, P-value for interface specificity, the number of salt bridges and the number of hydrogen bonds across the interface [64].

### 4.10. Protein-Ligand Docking

The molecular docking of identified and modeled defense related proteins namely, PR, PBPssh2p, PMSRc and the IAA protein with the ligand molecules β-1,3 glucan and chitin of fungi with using place an AutoDock 4.0 program [65]. The three dimensional structure of chitin (PDB ID: 3B9D) was obtained from the PDB and β-1,3 glucan reported [66]. The Lamarckian genetic algorithm was applied to perform automated molecular dockings with default parameters except for the number of runs. All torsions were allowed to rotate during docking and a grid map was created by an AutoGrid tool.

## 5. Conclusions

The application of novel proteomic and genetic engineering techniques will extend the possibilities of developing disease-resistant plants. Protein profiling using 2-DE studies revealed the accumulation of 21 differentially accumulated proteins in Foc-inoculated samples. Among these, PR protein, PMSRc, PBPssh2p and IAA proteins were predominantly related to the *Fusarium* wilt disease and validated through a qRT-PCR analysis. Homology modeling and *in silico* protein-protein docking of the modeled proteins with target fungal proteins can open new avenue for computer aided drug designing to better understand the potential proteins that acts against the molecules of the fungal pathogen. The protein-protein docking of all predicted structures with the fungal protein endopolygalacturonase resulted in a stable complex because of their positive ∆G^Diss^ values. Exploring protein-protein docking within defense related proteins and target fungal proteins can open the new avenues for computer-aided drug designing to better identify the potential proteins that act against the molecules of the fungal pathogen. The protein ligand docking study revealed that both the PBPssh2p and IAA proteins showed a better interaction with chitin and β-1, 3 glucan. With this data, the PBPssh2p and IAA proteins have been shown to have the potential to break down the fungal cell wall constituents of chitin and β-1, 3 glucan. This investigation accelerates the research in designing strategies to control the *Fusarium* wilt diseases of the banana plants.

## Figures and Tables

**Table 1:** Peptide mass fingerprinting analysis of differentially accumulated protein spots in *M. paradisiaca cv.* puttabale infected with *Fusarium oxysporum* f. sp*. cubense*.

Sl. No.	Protein Spot Number	Experimental Mw/pI	Theoretical Mw/pI	Protein Name by Mascot Search	Accession No.	Matched Peptide	Sequence Coverage %	Banana Genome Hub Hit Accession No./Name/Score/E-Value
1	1104	16,634/4.59	16,866.31/5.19	Class 10 pathogenesis-related protein 1	Q43560	6	44	ITC1587_Bchr3_P05906/pathogenesis-related protein/70/8 × 10^−13^
2	1401	25,720/4.81	126,342.70/8.05	Contains similarity to PHD-fingers domain proteins (*Arabidopsis thaliana*)	AAD03428	6	33	ITC1587_Bchr5_P13750/ring fyve phd zinc finger protein/137/4 × 10^−33^
3	2401	24,525/4.93	28,994.06/5.43	DHAR class glutathione transferase DHAR1 (*Populus trichocarpa*)	ADB11343	7	51	ITC1587_Bchr7_P20675/dehydroascorbate reductase/300/7 × 10^−82^
4	2403	25,368/5.27	42,346.20/5.52	Caffeine synthase 1 (*Triticum urartu*)	EMS67385	6	32	ITC1587_Bchr1_P01127/salicylate o-methyltransferase-like/102/2 × 10^−22^
5	3004	12,818/5.96	93,030.16/5.90	Sucrose synthase-like protein, partial (*Picea sitchensis*)	ADM74135	5	76	ITC1587_Bchr10_P30544/sucrose synthase/159/5 × 10^−40^
6	3103	16,247/5.77	16,029.46/5.78	Dynein heavy chain 3, partial (*Chlamydomonas reinhardtii*)	AAC49516	11	80	---
7	3205	17,303/5.73	17,034.50/5.19	Pathogenesis-related protein STH-21 (*Solanum tuberosum*)	P17641	6	69	ITC1587_Bchr3_P05907/pathogenesis-related protein/96/1 × 10^−20^
8	4205	16,972/6.91	16,298.12/6.91	Cadmium/zinc-transporting ATPase (*Theobroma cacao*)	EOY20393	3	43	---
9	4402	25,304/6.40	26,983.57/5.21	Putative ankyrin (ISS) (*Ostreococcus tauri*)	XP_003082621	5	37	ITC1587_Bchr9_P25841/26s proteasome non-atpase regulatory subunit/100/8 × 10^−22^
10	4403	23,397/6.90	48,277.56/8.62	Predicted ras-related protein RABB1c-like (*Solanum lycopersicum*)	XP_004243219	11	55	ITC1587_Bchr9_P28079/ras-related protein rabb1c-like/411/1 × 10^−115^
11	4503	33,478/6.55	40,800.83/6.42	Alcohol dehydrogenase (*Miscanthus sinensis var. formosanus*)	CAD56710	7	31	ITC1587_Bchr8_P24615/alcohol dehydrogenase 1/545/1 × 10^−155^
12	4505	29,333/6.16	58,925.24/7.15	Sec14p-like phosphatidylinositol transfer family protein isoform 1 (*Theobroma cacao*)	EOY22665	6	36	ITC1587_Bchr8_P21515/polyphosphoinositide binding protein ssh2p/308/3 × 10^-84^
13	5007	14,477/6.69	13,916.02/6.68	Albumin-1 D (*Pisum sativum)*	P62929	5	56	----
14	5302	18,806/6.82	27,368.97/8.73	Peptide methionine sulfoxide reductase B5-like (*Vitis vinifera*)	XP_002278920	5	29	ITC1587_Bchr6_P15995 peptide methionine sulfoxide reductase chlo/110/6 × 10^−25^
15	6001	13,806/7.14	142,006.47/5.50	Disease resistance protein I-2 (*Solanum lycopersicum*)	ACO52382	6	48	ITC1587_Bchr6_P18091 disease resistance rpp13-like protein 1-like/132/7 × 10^−32^
16	5303	23,215/6.96	105,978.42/8.56	Ras related protein Rab-2-A (Zea mays)	P49103	6	39	ITC1587_Bchr4_P08535/subtilisin-like protease/376/1× 10^−105^
17	4601	46,574/6.18	69,200.95/6.17	Unnamed protein product (*Arabidopsis thaliana*)	BAB02183	7	28	ITC1587_Bchr3_P05170 protein odr-4 homolog/140/2 × 10^−33^
18	7704	46,093/8.79	41,519.59/7.62	Predicted protein (*Bathycoccus prasinos*)	CCO18444	14	52	ITC1587_Bchr5_P12322 lrr repeats and ubiquitin-like domain-containing protein/110/1 × 10^−24^
19	4404	25,556/6.74	34,849.29/8.32	Auxin-responsive protein IAA17 (*Arabidopsis thaliana)*	NP_171921	15	69	ITC1587_Bchr2_P04450/auxin-responsive protein/203/1 × 10^−52^
20	1002	11,693/4.22	30,370.08/5.10	60S acidic ribosomal protein P2B (Zea mays)	NP_001105390	4	52	ITC1587_Bchr10_P29925 60s acidic ribosomal protein p2a/107/1 × 10^−24^
21	4101	15,239/5.76	52,570.94/7.71	LEAFY 1, partial (*Malus sieboldii*)	ADG85488	6	73	ITC1587_Bchr6_P16241 leafy-like protein/100/3 × 10^−22^

## Supplementary Materials

The supplementary materials are available online at www.mdpi.com/2227-7382/4/1/9/s1.

## Abbreviations

The following abbreviations are used in this manuscript:

Foc: *Fusarium oxysporum* f. sp. *Cubense*
ITS: Internal transcribed spacer
PBPssh2p: Polyphosphoinositide binding protein ssh2p
IAA: Indoleacetic acid-induced-like
PR: Pathogenesis related protein
PG: Endopolygalacturonase
PMSRc: Peptide methionine sulfoxide reductase chloroplastic-like protein
2-DE: Two-dimensional gel electrophoresis
FDR: False discovery rate
MALDI-TOF: Matrix-assisted laser desorption/ionization Time of Flight
Mw: Molecular weight
pI: Isoelectric point
qRT-PCR: Quantitative reverse transcription PCR
DRrpp13: disease resistance rpp13-like protein 1-like
PDB: Protein Data Bank
GDT-TS: Global Distance Test Total Score
RMSD: Root Mean Square Deviation
CASTp: Computed Atlas of Surface Topography of proteins
Rg: radius of gyration
RMSF: root mean square deviation
GRAMM-X: lobal Range Molecular Matching
