# Supplementary material for: Identification of Biomarkers for Resistance to Fusarium oxysporum f. sp. cubense Infection and in Silico Studies in Musa paradisiaca Cultivar Puttabale through Proteomic Approach
Source: Proteomes. 2016 Feb 24;4(1):9. doi: 10.3390/proteomes4010009 (PMC5217371; doi:10.3390/proteomes4010009)
Supplement: Supplementary file 1 [file proteomes-04-00009-s001.pdf]

proteomes

Supplementary Materials: Identification of Biomarkers for Resistance to *Fusarium oxysporum* f. sp. *cubense* Infection and *in Silico* Studies in *Musa paradisiaca* Cultivar Puttabale through Proteomic Approach

Venkatesh Ramu, Krishna Venkatarangaiah, Pradeepa Krishnappa, Santosh Kumar Shimoga Rajanna, Nagaraja Deeplanaik, Anup Chandra Pal and Kukkundoor Ramachandra Kini

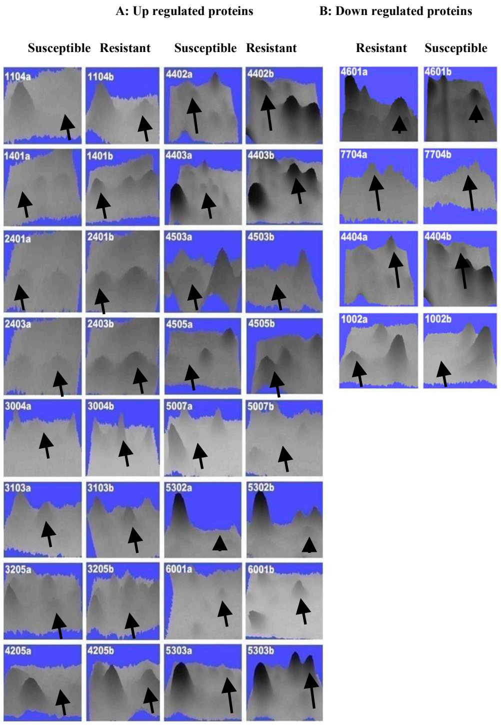

Figure S1. 3D images of resolved differentially expressed proteins. (**A**) Up regulated protein spots; (**B**) Down regulated protein spots. 1104—Pathogenesis-related protein; 1401—Ring fyve phd zinc finger protein; 2401—Dehydroascorbate reductase; 2403—Salicylate o-methyltransferase-like; 3004—Sucrose synthase; 3103—Dynein heavy chain; 3205—Pathogenesis-related protein; 4205—Cadmium/zinc-transporting atpase; 4402—26s proteasome non-atpase regulatory subunit; 4403—Ras-related protein rabb1c-like; 4503—Alcohol dehydrogenase 1; 4505—Polyphosphoinositide binding protein ssh2p; 5007—Albumin-1 D; 5302—Peptide methionine sulfoxide reductase chlo; 6001—Disease resistance rpp13-like protein 1-like; 5303—Subtilisin-like protease; 4601—Protein odr-4; 7704—Lrr repeats and ubiquitin-like domain-containing protein; 4404—Auxin-responsive protein; 1002—60s acidic ribosomal protein.

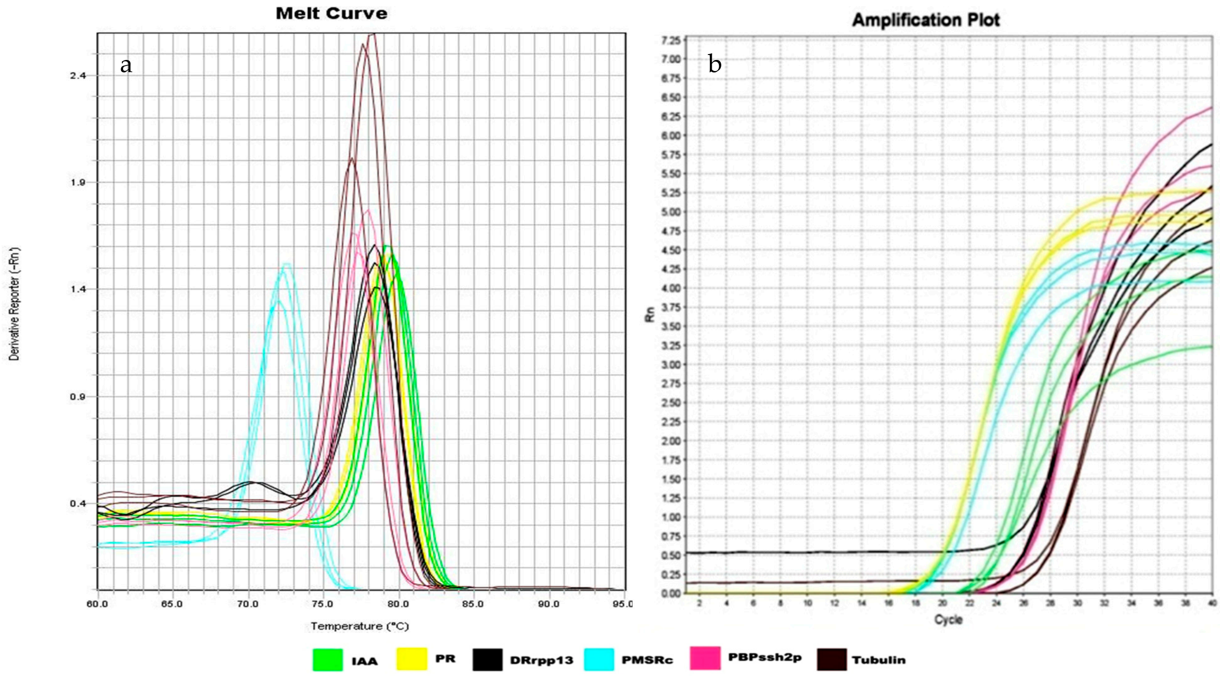

Figure S2. (**a**) Melting curve and (**b**) Amplification plot Specificity of the PCR reactions of PR, PBPssh2p, PMSRc, IAA, DRrpp13 gene.

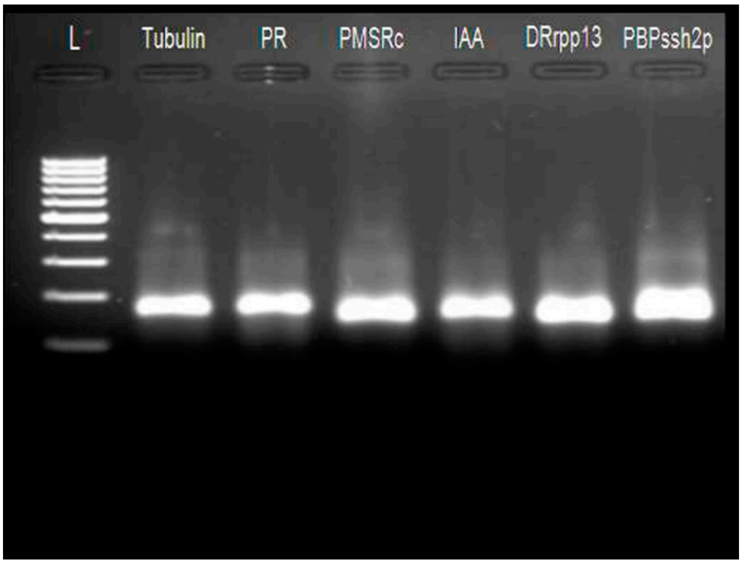

Figure S3. The specific amplicon size of Tubulin, PR, PMSRc, PBPssh2p, IAA, DRrpp13 gene on 3.5% agarose gel.

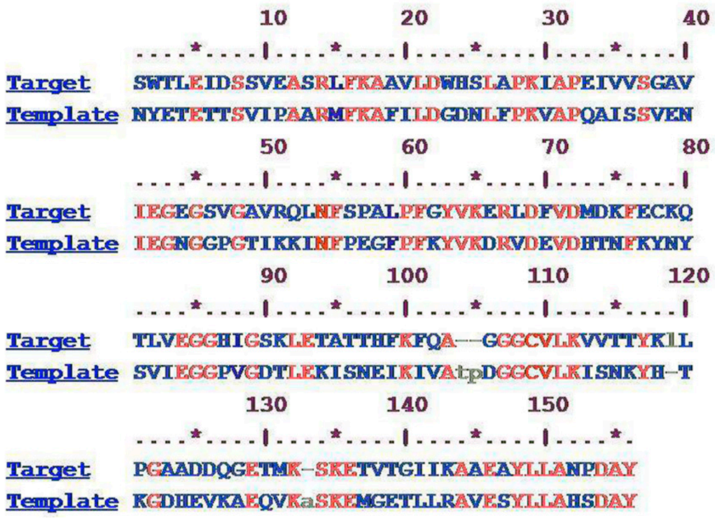

Figure S4. PR (*Musa* sps.) sequence with homologues Birch Pollen Allergen Bet V 1 (PDB-1FM4_A) shared 41% of identity. Conserved residues are represented by asterisk (*), gaps are represented by hyphen (–), weak conserved amino acids are denoted by (.), respectively.

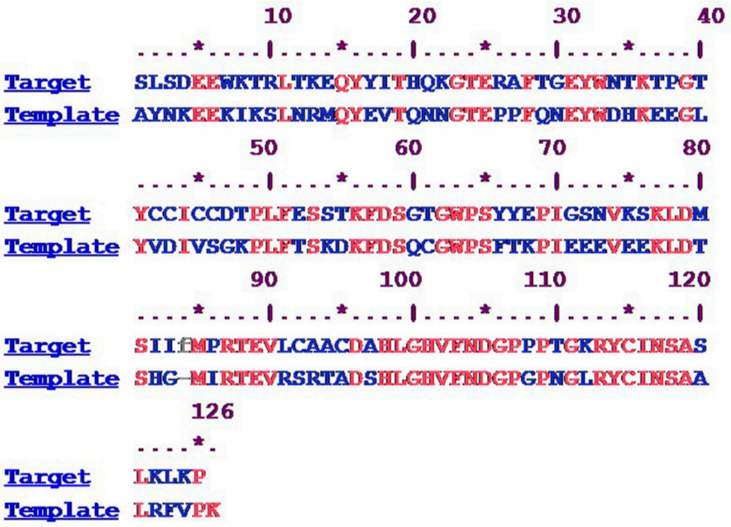

Figure S5. Alignment of PMSRc (Musa sps.) sequence with homologues Peptide Methionine Sulfoxide Reductase Msrb (*Bacillus Subtilis*; PDB-2KZN_A) shared 51% of identity. Conserved residues are represented by asterisk (*), gaps are represented by hyphen (–), weak conserved amino acids are denoted by (.), respectively.

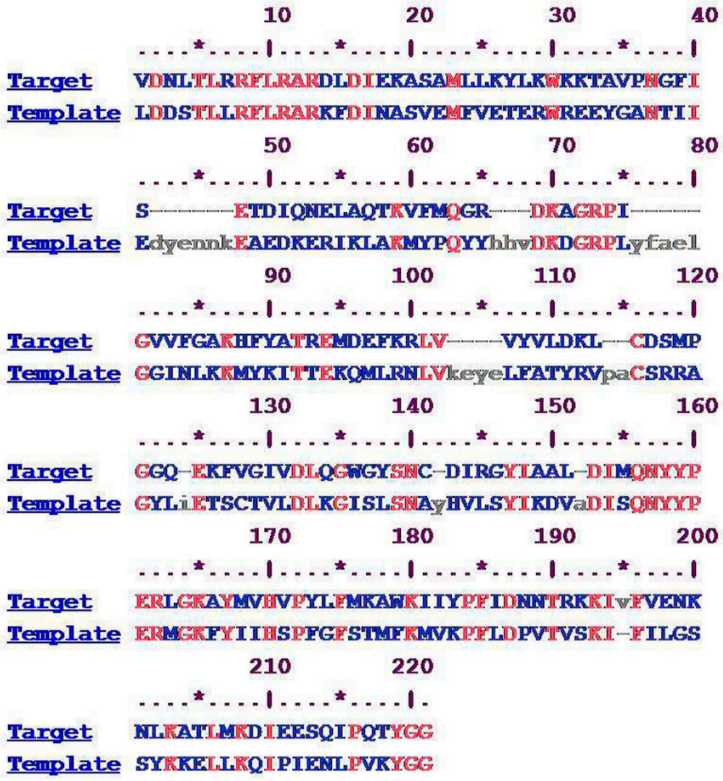

Figure S6. Alignment of PBPssh2p (Musa sps.) sequence with homologues Functional Phosphatidylinositol Transfer Protein from A Pseudo-Sec14 Scaffold (PDB-3Q8G_A) By Directed Evolution shared 35% of identity. Conserved residues are represented by asterisk (*), gaps are represented by hyphen (–), weak conserved amino acids are denoted by (.), respectively.

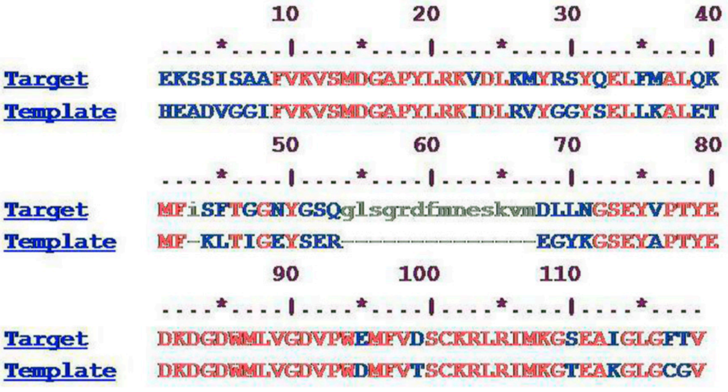

Figure S7. Alignment of IAA (*Musa* sps.) sequence with homologues Aux/iaa Transcription Factor Ps-iaa4 From Pea (*Pisum sativum*, PDB-2M1M_A) shared 57% of identity. Conserved residues are represented by asterisk (*), gaps are represented by hyphen (–), weak conserved amino acids are denoted by (.), respectively.

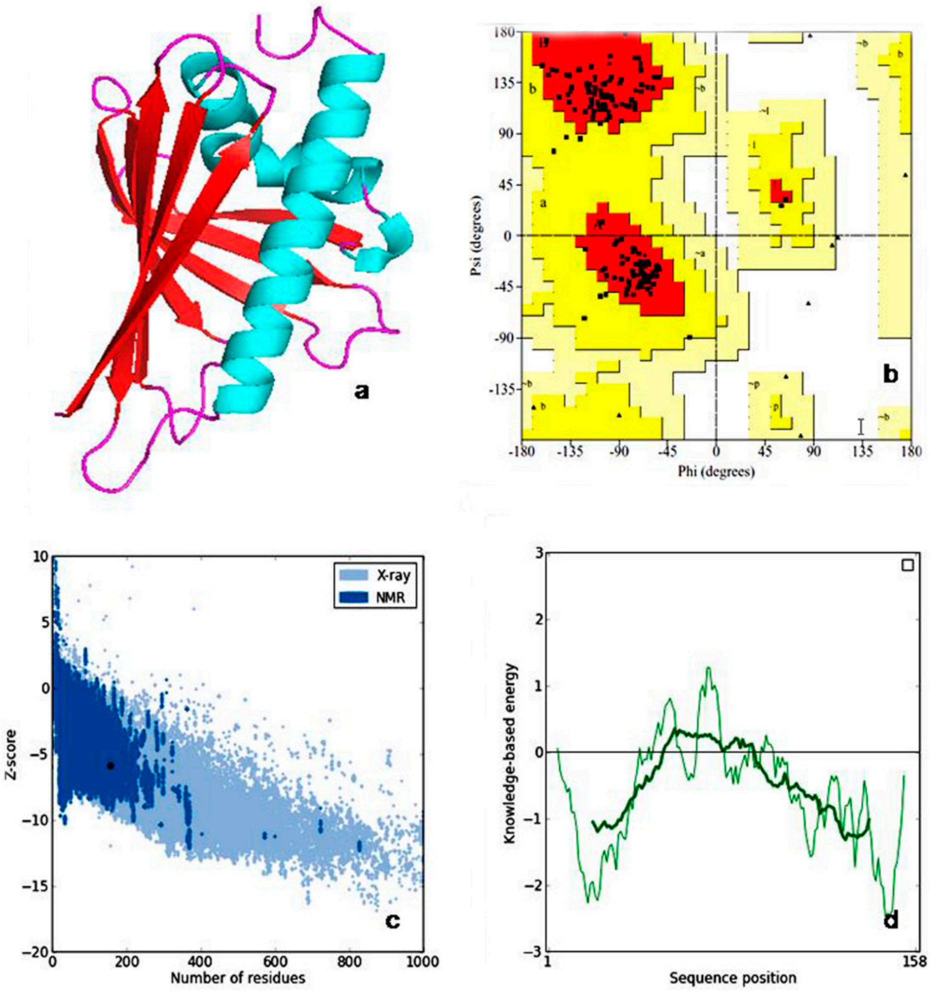

Figure S8. The Homology modeling and structure validation of PR protein. (**a**) Homology modeling; (**b**) Ramachandra plot; (**c**) The proSA surface energy z score plot; (**d**) Energy profile of proSA plot.

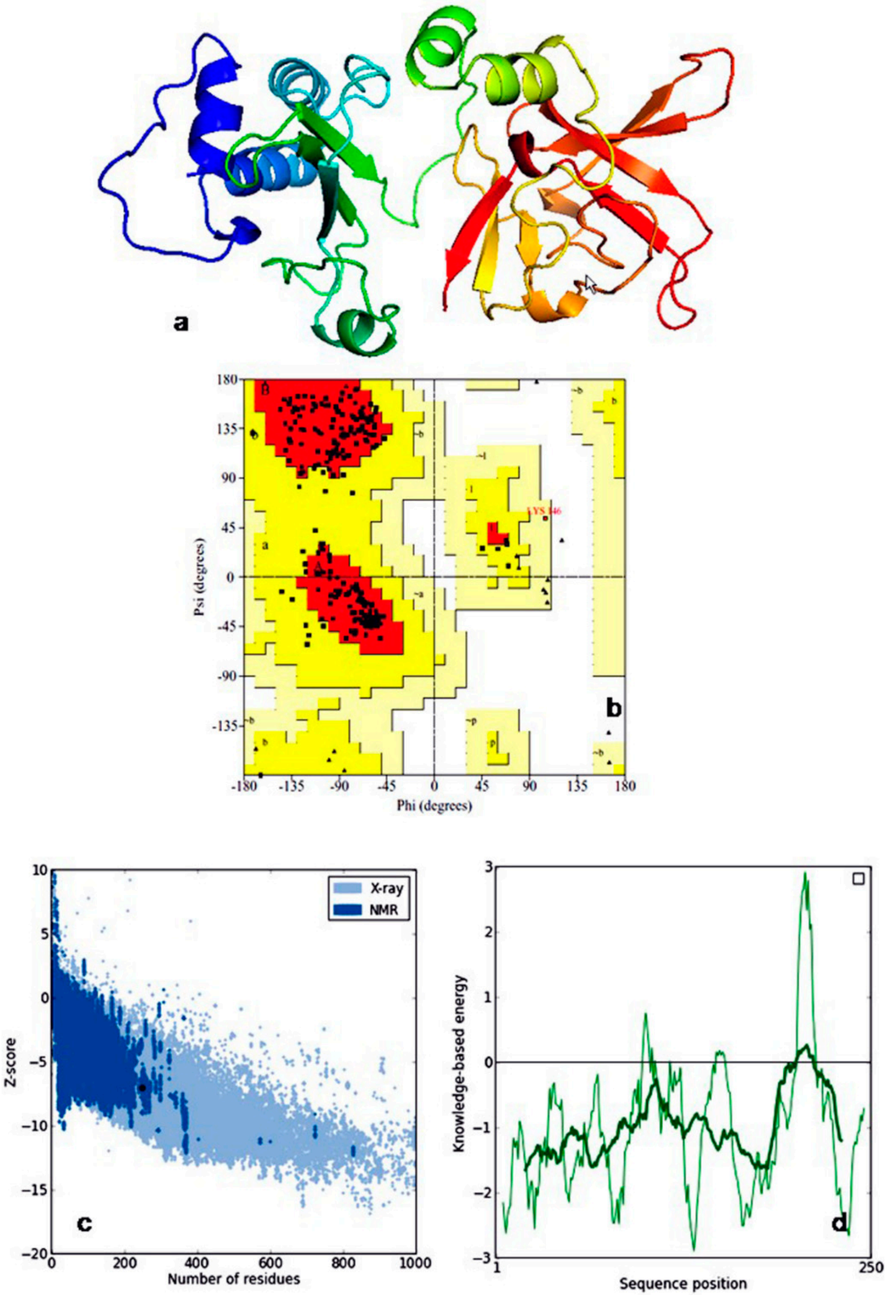

Figure S9. The Homology modeling and structure validation of PMSRc protein. (**a**) Homology modeling; (**b**) Ramachandra plot; (**c**) The proSA surface energy z score plot; (**d**) Energy profile of proSA plot.

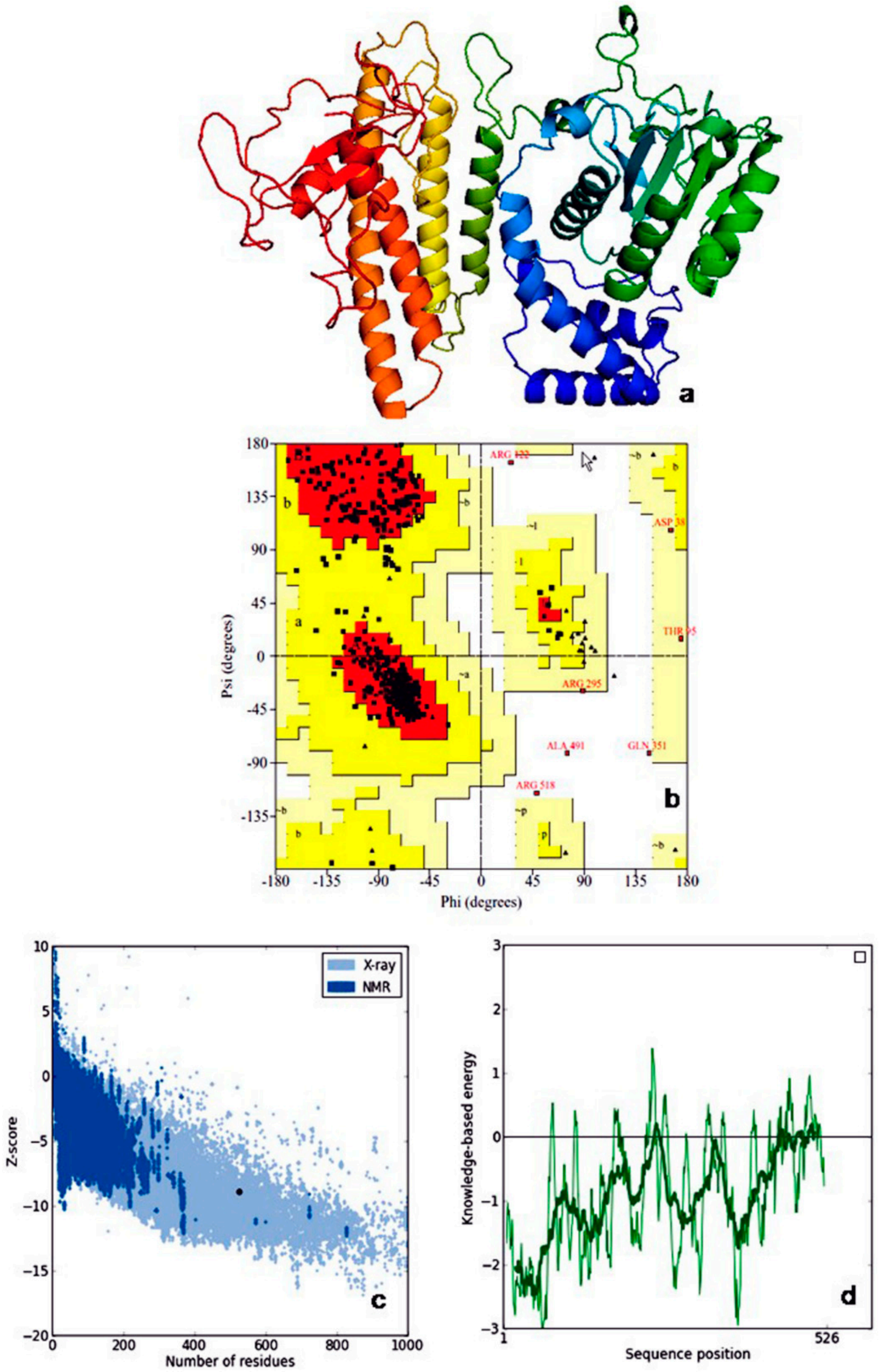

Figure S10. The Homology modeling and structure validation of PBPssh2p protein. (**a**) Homology modeling; (**b**) Ramachandra plot; (**c**) The proSA surface energy z score plot; (**d**) Energy profile of proSA plot.

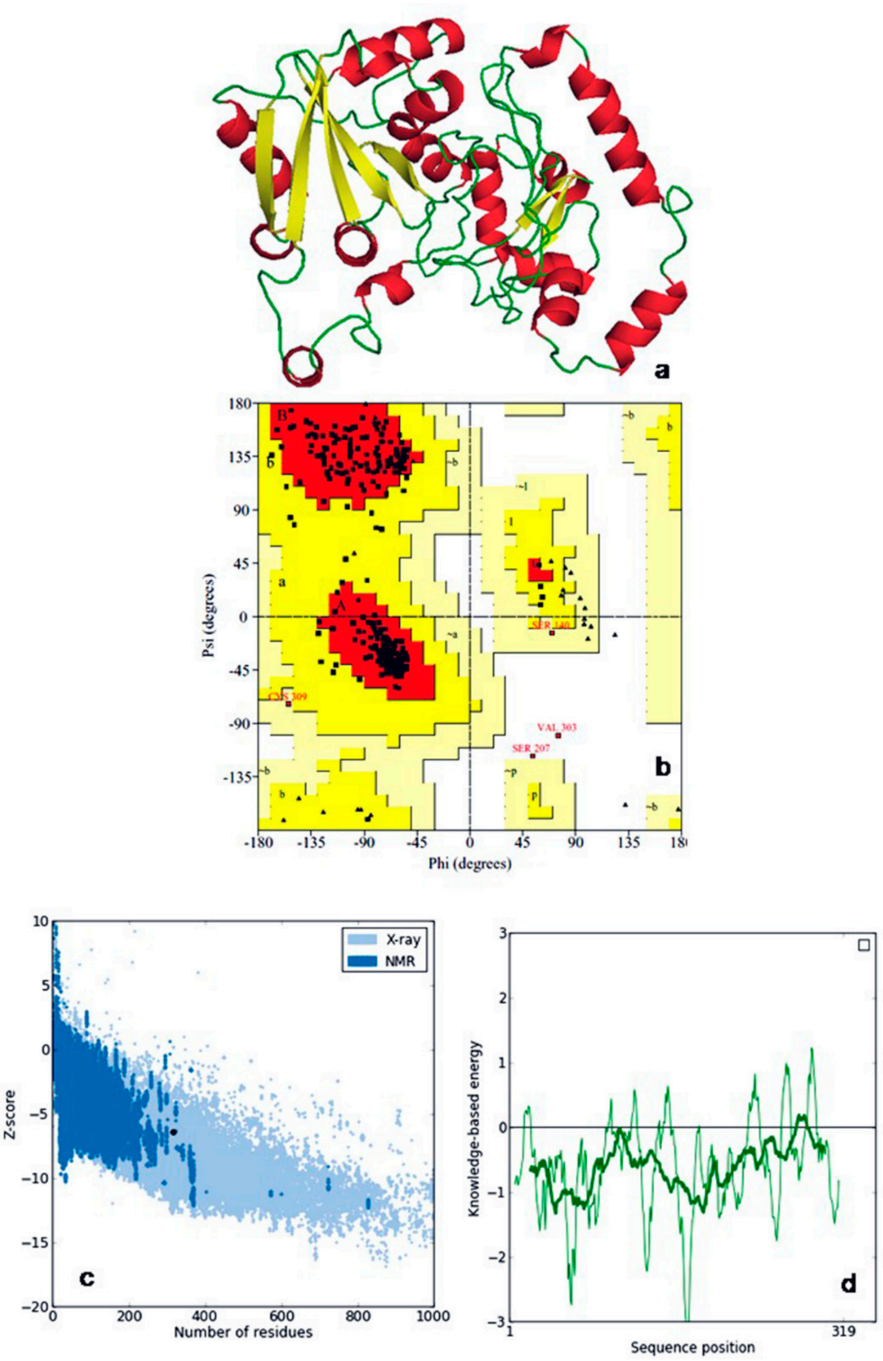

Figure S11. The Homology modeling and structure validation of IAA protein. (**a**) Homology modeling; (**b**) Ramachandra plot; (**c**) The proSA surface energy z score plot; (**d**) Energy profile of proSA plot.

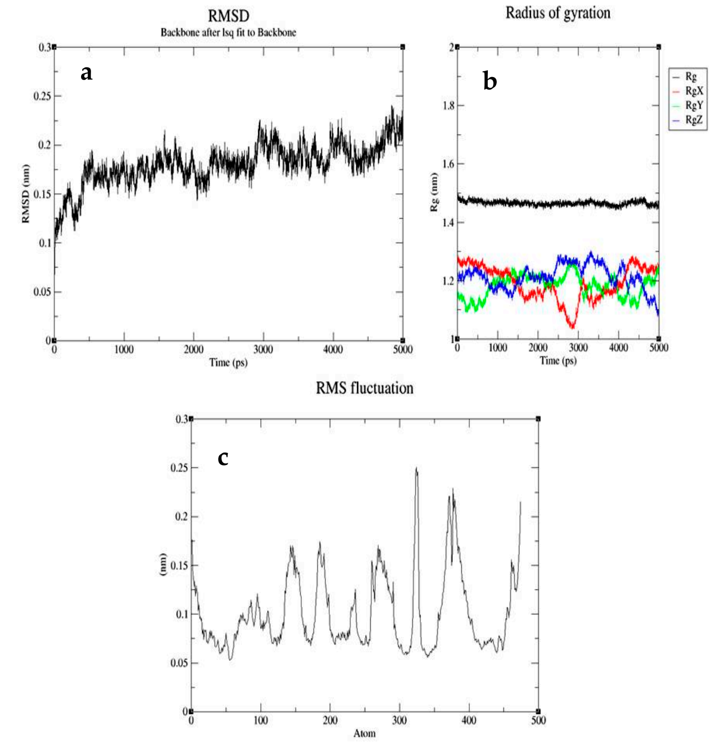

Figure S12. Molecular dynamic simulation of PR protein. (**a**) RMSD plot at 10 ns timescale; (**b**) Radius of gyration plot for compactness of structure; (**c**) RMSF plot for fluctuation in backbone residues.

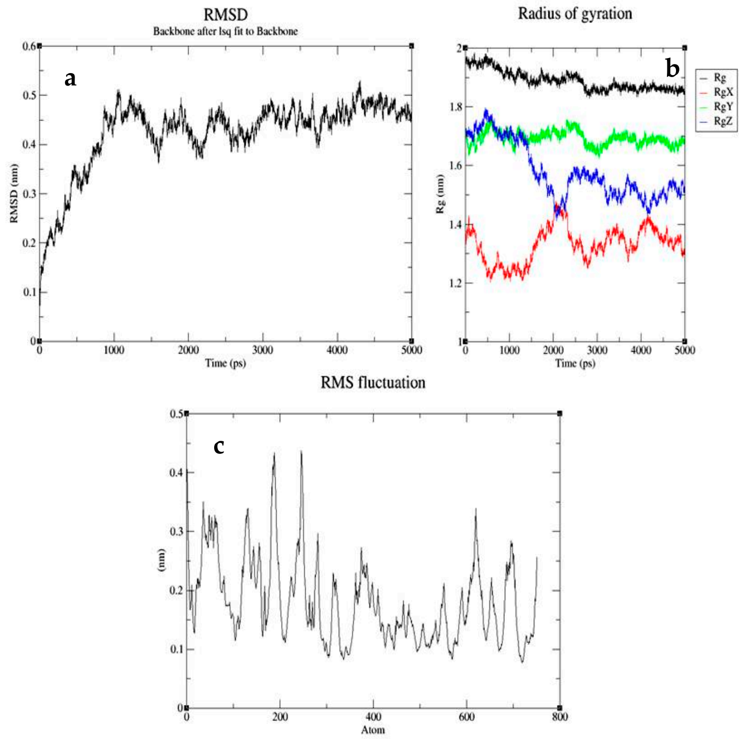

Figure S13. Molecular dynamic simulation of PMSRc protein. (**a**) RMSD plot at 10 ns timescale; (**b**) Radius of gyration plot for compactness of structure; (**c**) RMSF plot for fluctuation in backbone residues.

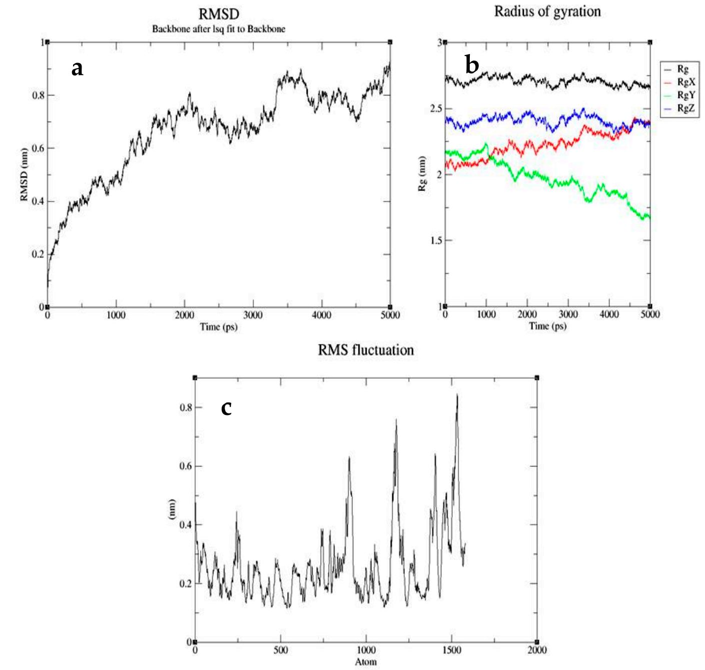

Figure S14. Molecular dynamic simulation of PBPssh2p protein. (**a**) RMSD plot at 10 ns timescale; (**b**) Radius of gyration plot for compactness of structure; (**c**) RMSF plot for fluctuation in backbone residues.

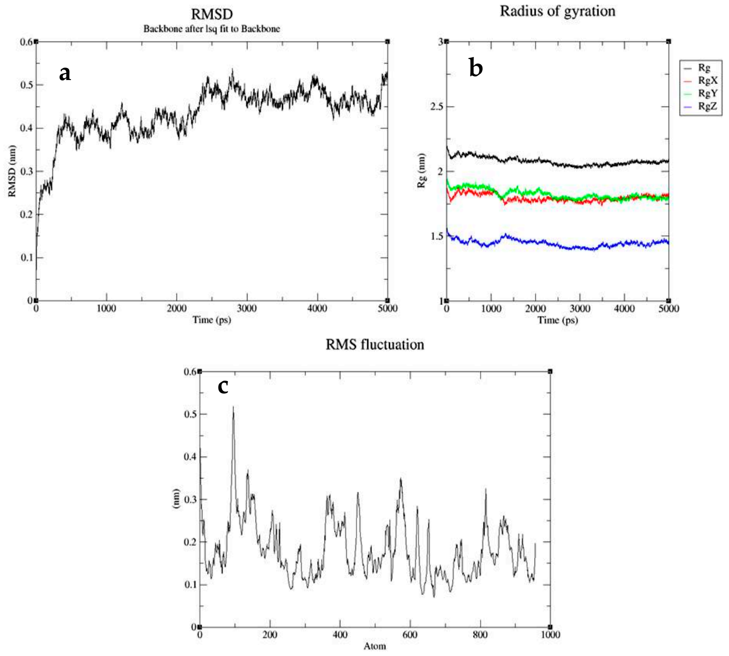

Figure S15. Molecular dynamic simulation of IAA protein. (**a**) RMSD plot at 10 ns timescale; (**b**) Radius of gyration plot for compactness of structure; (**c**) RMSF plot for fluctuation in backbone residues.

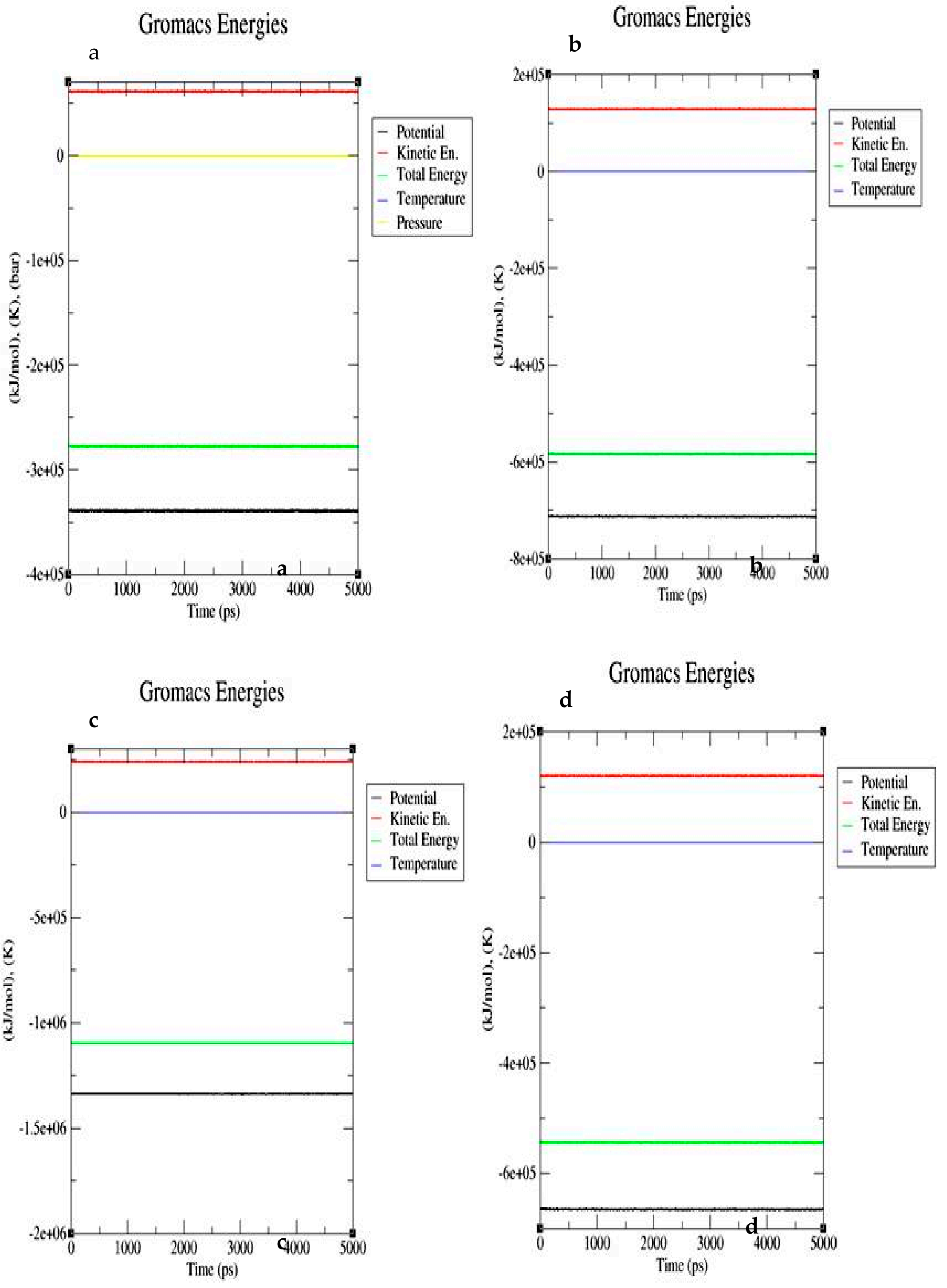

Figure S16. Total potential energy of the modeled proteins viz., (**a**) PR; (**b**) PMSRc; (**c**) PBPssh2p; (**d**) IAA.

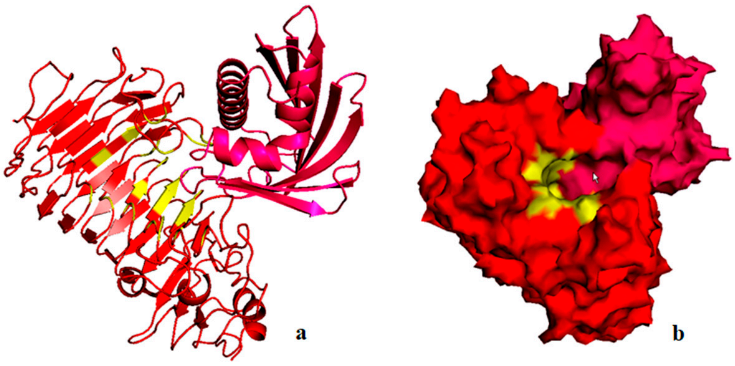

Figure S17. The protein-protein docking of PR protein to PG molecule using Gramm-X server. The PG molecule highlighted with tv_red, active pocket colored with yellow color and PR proetin are highlighted with hotpink color. (**a**) Cartoon view; (**b**) Molecular surface view.

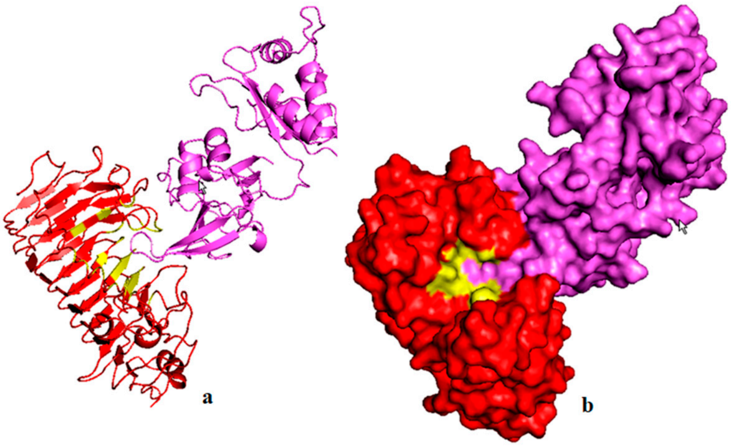

Figure S18. Protein-protein docking of PMSRc protein with PG molecule. The PG molecule highlighted with tv_red, active pocket colored with yellow and the PMSRc protein highlighted with lightmagenta color. (**a**) Cartoon view; (**b**) Molecular surface view.

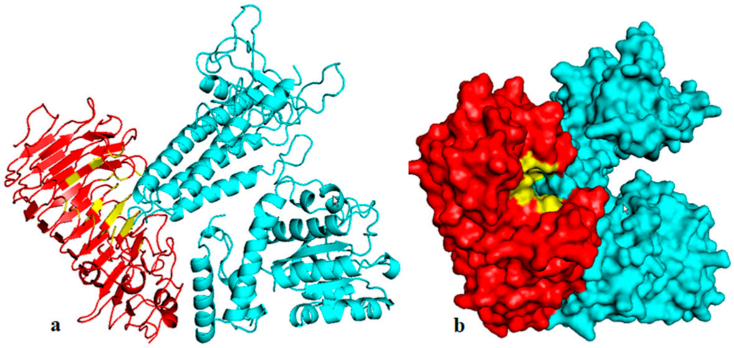

Figure S19. Protein-protein docking of PBPssh2p protein to PG molecule. The PG molecule highlighted with tv_red, active pocket colored with yellow and the PBPssh2p protein highlighted with cyans color. (**a**) Cartoon view; (**b**) Molecular surface view.

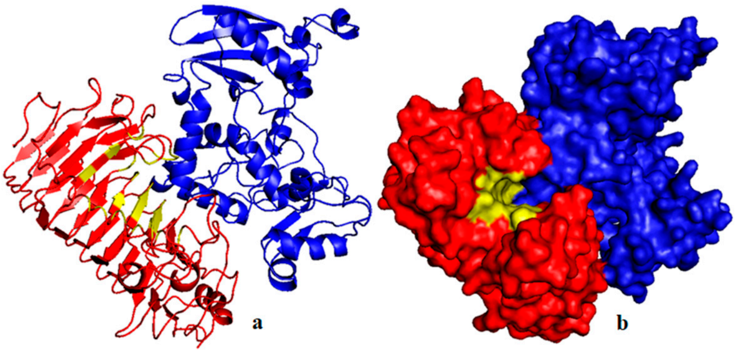

Figure S20. Protein-protein docking of IAA protein to PG molecule. The PG molecule highlighted with tv_red, active pocket colored with yellow and IAA protein highlighted with blue color. (**a**) Cartoon view; (**b**) Molecular surface view.

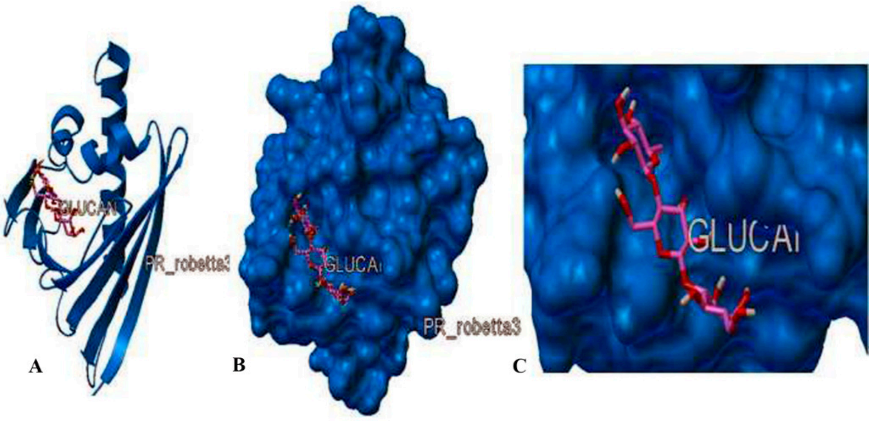

Figure S21. Docking of PR protein with Glucan compound. (**A**) Ribbon structure of PR and Glucan compound; (**B**) Molecular surface view of PR and Glucan compound; (**C**) Glucan compound compactly inserted into the active pocket of PR protein.

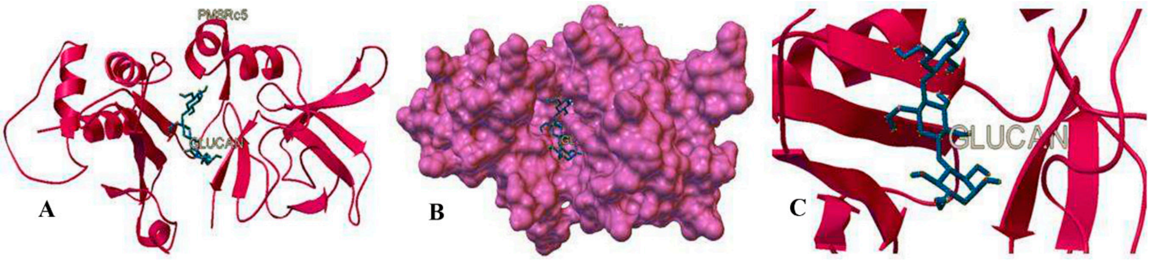

Figure S22. Docking of PMSRc protein with Glucan compound. (**A**) Ribbon structure of PMSRc and Glucan compound; (**B**) Molecular surface view of PMSRc and Glucan compound; (**C**) Glucan compound compactly inserted into the active pocket of PMSRc protein.

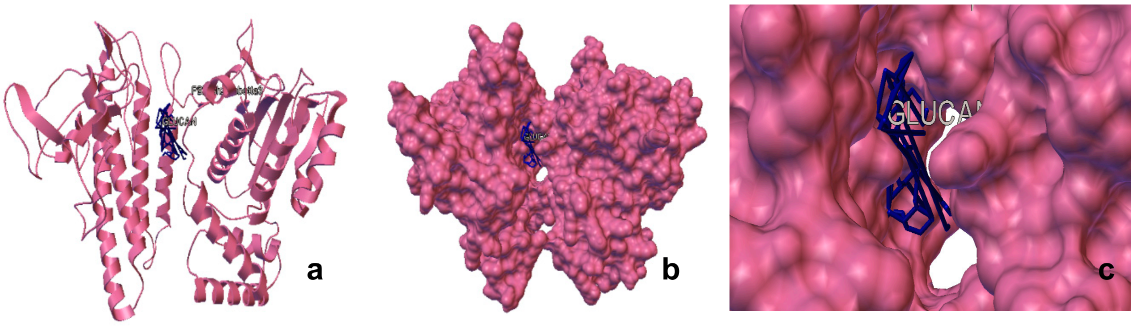

Figure S23. Docking of PBPssh2p protein with Glucan compound. (**a**) Ribbon structure of PBPssh2p and Glucan compound; (**b**) Molecular surface view of PBPssh2p and Glucan compound; (**c**) Glucan compound compactly inserted into the active pocket of PBPssh2p protein.

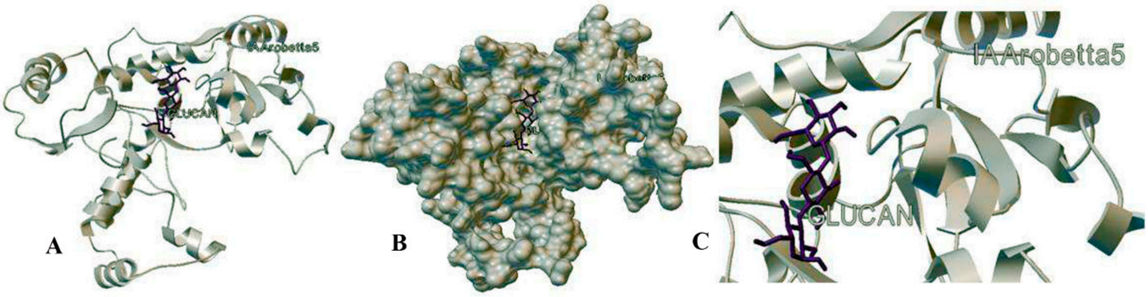

Figure S24. Docking of IAA protein with Glucan compound. (**A**) Ribbon structure of IAA and Glucan compound; (**B**) Molecular surface view of PBPssh2p and Glucan compound; (**C**) Glucan compound compactly inserted into the active pocket of IAA protein.

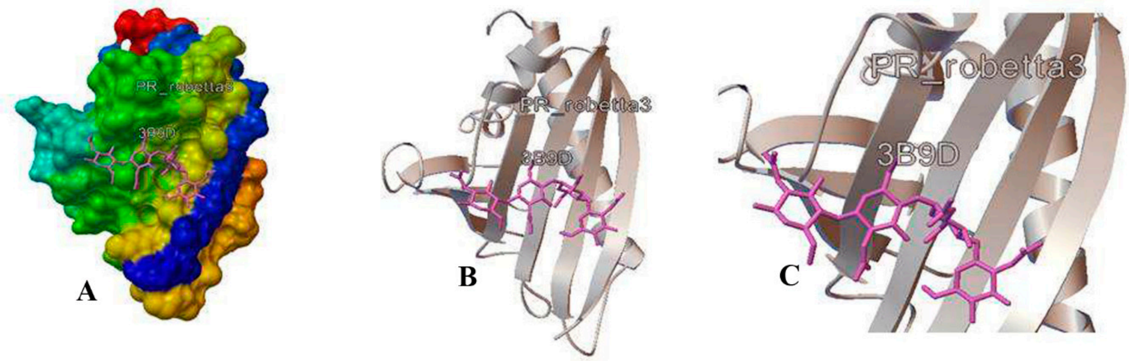

Figure S25. Docking of PR protein with chitin substrate. (**A**) Molecular surface view of PR and chitin substrate; (**B**) Ribbon structure of PR and Glucan substrate; (**C**) Chitin substrate compactly inserted into the active pocket of PR protein.

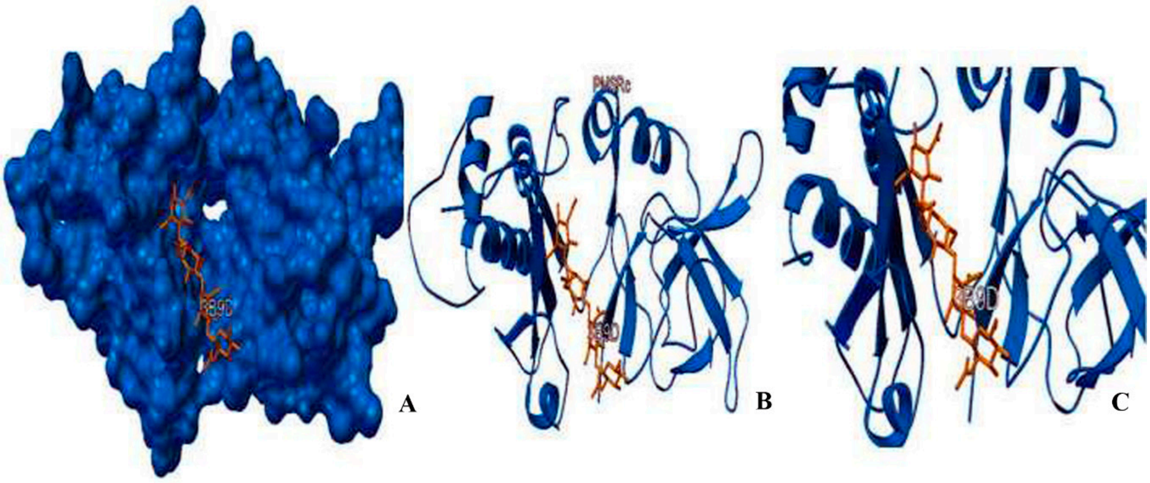

Figure S26. Docking of PMSRc protein with chitin substrate. (**A**) Molecular surface view of PMSRc and chitin substrate; (**B**) Ribbon structure of PMSRc and Glucan substrate; (**C**) Chitin substrate compactly inserted into the active pocket of PMSRc protein.

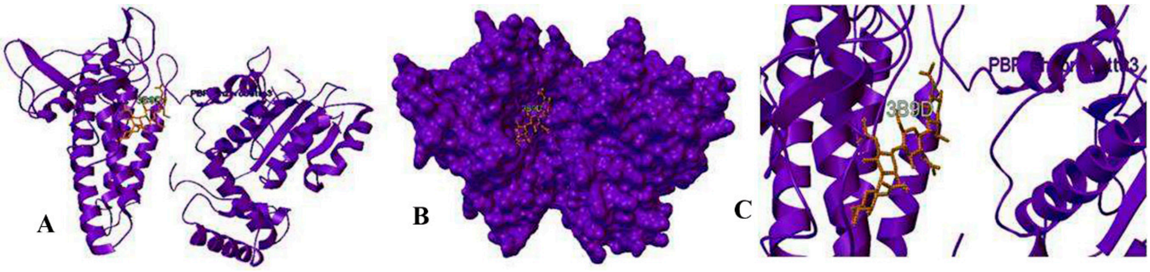

Figure S27. Docking of PBPssh2p protein with chitin substrate. (**A**) Ribbon structure of PBPssh2p and Glucan substrate; (**B**) Molecular surface view of PBPssh2p and chitin substrate; (**C**) Chitin substrate compactly inserted into the active pocket of PBPssh2p protein.

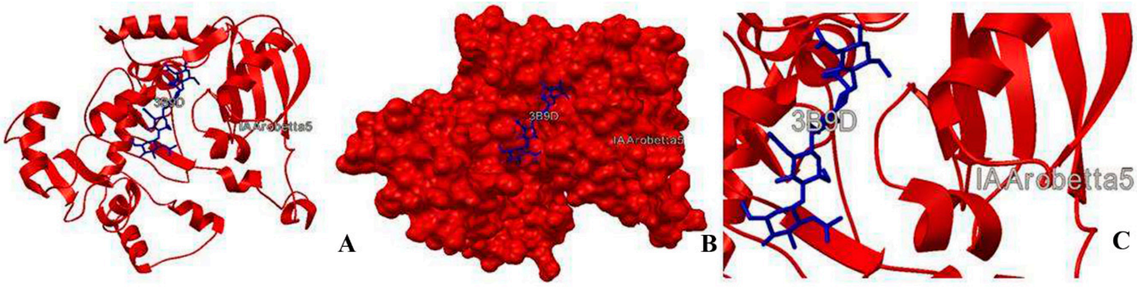

Figure S28. Docking of IAA protein with chitin substrate. (**A**) Ribbon structure of IAA and Glucan substrate; (**B**) Molecular surface view of IAA and chitin substrate; (**C**) Chitin substrate compactly inserted into the active pocket of IAA protein.

Table S1. The PDquest software analysis of differentially accumulated proteins in Foc infected leaves sample.

SL. NO.	SSP	Mr	pI	co	Ratio	tr	Ratio
1	1402	38.01	4.5	396.5	1		0.01
2	2702	70.6	4.7	1146.6	1	67.8	0.06
3	6103	21.72	7.49	1343.7	1	166.6	0.12
4	6101	21.14	7.13	1520.2	1	254.5	0.17
5	7602	47.12	7.78	812.2	1	141.7	0.17
6	6801	78.01	7.01	4905.2	1	1046.2	0.21
7	1002	18.9	3.9	1291.83	1	332.2	0.25
8	4402	38.25	6.18	2238.4	1	575.2	0.26
9	7704	60.71	8.23	3732.4	1	995.4	0.27
10	4601	57.16	6.01	11,968.1	1	3390.8	0.28
11	7802	71.03	7.93	2616	1	924.8	0.35
12	6104	20.61	7.52	1260.8	1	518.2	0.41
13	4101	22.92	5.78	1667.5	1	692.1	0.42
14	4503	45.25	6.25	3731.4	1	1553.6	0.42
15	5501	41.65	6.46	2192	1	1037.8	0.47
16	2602	49.32	4.85	3789.6	1	1861.9	0.49
17	4801	95.06	6.05	6323.8	1	3115.7	0.49
18	5701	70.36	6.56	67,127	1	42,162.7	0.63
19	5602	47.22	6.66	1664.6	1	1097.2	0.66
20	3203	26.19	5.54	730.5	1	500.8	0.69
21	2705	65.27	5.08	10,566.8	1	8457.8	0.8
22	5601	51.63	6.5	8064.3	1	7172.3	0.89
23	8601	52.13	8.4	3039.8	1	2761.5	0.91
24	4303	31.92	6.11	7194	1	7422.1	1.03
25	1204	27.99	4.25	496.6	1	574.5	1.16
26	3603	47.43	5.69	4655.8	1	5818.4	1.25
27	2401	38.1	4.7	729	1	946	1.29
28	7601	53.28	7.64	1564.3	1	2039.6	1.3
29	3602	47.82	5.54	2492.6	1	3344.8	1.34
30	4404	39.31	6.41	1175.3	1	1671.6	1.42
31	4204	23.96	6.17	996.1	1	1478.9	1.48
32	2501	41.23	4.83	1292.4	1	1946.8	1.51
33	2403	37.9	5	1345.5	1	2052.87	1.52
34	2701	61.16	4.81	1645.2	1	2600.7	1.58
35	6702	60.82	7.46	1849.7	1	2954.3	1.6
36	4102	21.8	5.85	575.1	1	940.5	1.64
37	4202	24.94	6.05	2515.8	1	4134.1	1.64
38	1206	24.35	4.48	1179.9	1	1954.2	1.66
39	4602	49.4	6.04	3691	1	6791.5	1.84
40	3605	59.36	5.32	3556.5	1	7040.2	1.98
41	3101	20.45	5.41	591.6	1	1171.6	1.98
42	2405	40.29	5.06	687.9	1	1376.9	2
43	1401	37.6	4.5	365.91	1	763.08	2.09
44	3205	24.89	5.72	3178.9	1	6697.5	2.11
45	2302	30.71	5.03	2201	1	4645	2.11
46	5302	29.12	6.55	811.7	1	1783.7	2.2
47	5304	31.82	6.59	802.1	1	1830.3	2.28
48	7701	62.35	7.63	1738	1	3985.5	2.29
49	4205	24.36	6.39	2142.5	1	4966.6	2.32
50	3804	98.99	5.61	3006.7	1	7322.5	2.44

Table 1. *Cont.*

SL. NO.	SSP	Mr	pI	co	Ratio	tr	Ratio
51	3201	28.92	5.4	1704.4	1	4673.6	2.74
52	4304	29.38	6.19	299.3	1	884	2.95
53	5003	18.12	6.95	212.3	1	629.5	2.97
54	4701	64.87	6.03	1966.4	1	6018.9	3.06
55	5303	35.29	6.55	1056.3	1	3341.8	3.16
56	2101	22.68	4.67	1834.1	1	6242.1	3.4
57	4403	35.34	6.4	716.7	1	2500.1	3.49
58	6001	18.99	7.18	348.7	1	1274.1	3.65
59	6502	42.54	7.13	238.8	1	1321.8	5.54
60	1104	22.37	4.37	210.2	1	1264.5	6.01
61	6202	23.75	7	314.6	1	2218	7.05
62	5603	51.41	6.73	1286.6	1	11,149.9	8.67
63	1008	16.96	4.42	64.2	1	581.9	9.06
64	2201	28.14	4.88	325.6	1	3237	9.94
65	5007	18.24	6.64	45.2	1	520.9	11.52
66	3004	18.26	5.5	39.5	1	1280.8	32.4
67	4505	43.81	6.37	33.4	1	1484.1	44.44
68	3103	22.14	5.53		1	858.6	200.65
69	5605	53.13	6.92		1	1467.4	342.94
70	4305	35.15	6.21		1	1590.2	371.65

Highlighted proteins spots are used for MALDI TOF analysis.

Table S2. Significance level correction by Benjamini-Hochberg FDR method.

Sl. No.	SSP No.	p	q*	p < q*
1	5303	0.0000	0.0023	TRUE
2	1002	0.0000	0.0045	TRUE
3	3004	0.0000	0.0068	TRUE
4	4503	0.0043	0.0114	TRUE
5	4402	0.0084	0.0136	TRUE
6	5302	0.0123	0.0159	TRUE
7	6001	0.0132	0.0182	TRUE
8	4601	0.0161	0.0227	TRUE
9	4404	0.0177	0.0250	TRUE
10	7704	0.0222	0.0273	TRUE
11	3205	0.0235	0.0295	TRUE
12	1104	0.0258	0.0318	TRUE
13	4505	0.0273	0.0341	TRUE
14	4403	0.0379	0.0364	FALSE
15	4205	0.0417	0.0386	FALSE
16	1401	0.0746	0.0409	FALSE
17	5007	0.0805	0.0432	FALSE
18	2403	0.0894	0.0455	FALSE
19	4101	0.1552	0.0477	FALSE
20	2401	0.2576	0.0500	FALSE

The Benjamini-Hochberg's FDR (q*) for the *p*-value of a t-test were calculated as [(i/m) q], where "m" is the number of *p*-values of the given dataset. The hypothesis for a given test is considered truly significant, *i.e.*, significantly rejects the null hypothesis only if its *p*-value is equal or less than that of Benjamini-Hochberg's correction (q*), *i.e.*, p < q*. The null hypothesis is that there is no significant difference in the protein abundance for a protein spot between control and treated samples.

Table S3. Analysis of the Interface of protein-protein docking of PR with PG using GRAMM X server.

Sl. No.	Surface Area, Å²	Buried Area, Å²	ΔG^int^ kcal/M	ΔG^diss^ kcal/M	No. of HB and HBE kJ/mol	No. of SB	No. of HI within 5 Å	EE kJ/mol	vWE kJ/mol	TSE kJ/mol	II within 6 Å	*p*-Value
01	19,272.1	2082.5	−11.6	2.1	6/1.14	1	2	51.5411	−31.3806	21.3004	1	0.209
02	19,492.4	1871.5	−6.5	−2.9	6/1.52	1	4	−0.67573	168.76	169.604	3	0.441
03	19,186.6	2171.4	−11.0	1.5	6/−5.72	1	7	25.2598	372.17	391.71	3	0.232
04	19,386.7	1971.9	−9.9	−0.5	4/−0.06	1	8	27.0203	−86.9892	−60.0289	1	0.271
05	19,679.0	1687.4	−7.6	−0.7	9/5.24	0	3	89.8989	1.47751	96.6164	2	0.330
06	19,384.9	1993.4	−9.2	0.7	8/21.17	2	3	6.54538	−21.9435	5.77187	2	0.281
07	19,895.2	1468.2	−8.5	0.6	9/10.49	2	3	111.712	58.9064	181.108	2	0.241
08	19,793.5	1576.2	−7.5	−1.6	7/0	0	5	33.4965	−97.0389	−63.5424	2	0.307
09	20,090.0	1278.1	−4.9	−4.2	7/0.5	0	2	90.2116	−120.028	−29.3162	1	0.431
10	19,218.4	2140.5	−5.8	−3.8	6/7.0	1	−	78.1799	355.868	441.047	1	0.468

ΔG^{int}: solvation Free energy gain upon formation of the assembly; ΔG^{diss}: Free energy of assembly dissociation; HB: Hydrogen bonds across the interface; HBE: Hydrogen Bond Energy; SB: Salt bridges across the interface; HI: Hydrophobic Interactions; EE: Electrostatic Energy; vWE: van der Waals Energy; TSE: Total Stabilizing Energy; II: Ionic Interactions.

Table S4. Analysis of the Interface of protein-protein docking of PMSRc with PG using GRAMM X server.

Sl. No.	Surface Area, Å²	Buried Area, Å²	ΔG^int^ kcal/M	ΔG^diss^ kcal/M	No. of HB and HBE kJ/mol	No. of SB	No. of HI within 5 Å	EE kJ/mol	vWE kJ/mol	TSE kJ/mol	II within 6 Å	*p*-Value
01	25,937.4	1678.1	−7.9	−2.2	6/−2.28	0	1	40.6982	−112.718	−74.2994	0	0.183
02	25,624.3	2001.0	−2.3	−6.7	9/10.31	0	0	19.3266	−129.962	−100.325	0	0.526
03	25,658.5	1969.6	−4.5	−3.2	12/19.07	0	1	22.3422	−104.633	−63.2209	0	0.451
04	25,613.5	2004.5	−8.8	1.5	13/5.09	0	6	23.9878	−91.8147	−62.7369	1	0.109
05	25,454.4	2174.4	−9.2	−0.3	8/1.3	0	3	22.7511	73.5451	97.5962	0	0.095
06	25,253.3	2362.6	−4.8	−1.6	15/5.31	0	0	87.4518	54.284	147.046	1	0.416
07	25,440.3	2193.3	−6.7	−0.9	12/−5.65	0	1	36.8482	7.90308	39.1013	0	0.343
08	25,649.7	1978.5	−11.3	0.7	5/2.12	1	6	−8.92994	−53.9826	−60.7926	1	0.109
09	25,247.2	2391.8	−6.7	−0.3	13/3.76	2	2	−5.02864	−24.9429	−26.2115	5	0.361
10	25,437.7	2200.3	−5.4	−5.2	5/3.73	1	5	11.611	327.106	342.447	1	0.428

ΔG^{int}: solvation Free energy gain upon formation of the assembly; ΔG^{diss}: Free energy of assembly dissociation; HB: Hydrogen bonds across the interface; HBE: Hydrogen Bond Energy; SB: Salt bridges across the interface; HI: Hydrophobic Interactions; EE: Electrostatic Energy; vWE: van der Waals Energy; TSE: Total Stabilizing Energy; II: Ionic Interactions.

Table S5. Analysis of the Interface of protein-protein docking of PBPssh2p with PG using GRAMM X server.

Sl. No.	Surface Area, Å²	Buried Area, Å²	ΔG^int kcal/M	ΔG^diss kcal/M	No. of HB and HBE kJ/mol	No. of SB	No. of HI within 5 Å	EE kJ/mol	vWE kJ/mol	TSE kJ/mol	II within 6 Å	*p*-Value
01	42,871.7	2264.2	−8.0	−3.0	5/1.98	4	9	−27.8272	−51.3615	−77.2087	3	0.276
02	42,795.4	2356.4	−15.2	4.0	6/−0.6	0	3	32.789	432.02	464.209	1	0.053
03	42,867.6	2286.9	−9.7	−0.4	8/7.83	1	5	−14.2002	−162.853	−169.223	1	0.209
04	42,339.8	2805.6	−1.5	−9.3	7/11.7	1	4	−28.3642	−2.7757	−19.4399	4	0.686
05	42,589.0	2563.0	−4.2	−4.5	12/7.58	0	1	134.355	−19.4571	122.478	4	0.564
06	43,491.6	1653.1	−7.3	−2.7	8/9.57	1	2	61.165	287.763	358.498	1	0.244
07	42,078.8	3075.2	−8.8	−0.8	10/24.53	0	5	41.6887	68.2277	134.446	3	0.326
08	42,188.2	2954.6	−3.9	−4.9	11/25.84	2	3	59.8287	217.661	303.33	3	0.512
09	42,501.4	2639.4	−12.8	−3.1	9/−1.4	1	8	2.03713	39.4191	40.0562	2	0.129
10	42,364.9	2770.8	−6.1	−4.3	8/8.96	0	4	151.286	190.499	350.745	2	0.429

ΔG^{int}: solvation Free energy gain upon formation of the assembly; ΔG^{diss}: Free energy of assembly dissociation; HB: Hydrogen bonds across the interface; HBE: Hydrogen Bond Energy; SB: Salt bridges across the interface; HI: Hydrophobic Interactions; EE: Electrostatic Energy; vWE: van der Waals Energy; TSE: Total Stabilizing Energy; II: Ionic Interactions.

Table S6. Analysis of the Interface of protein-protein docking of IAA with PG using GRAMM X server.

Sl. No.	Surface Area, Å²	Buried Area, Å²	ΔG^int kcal/M	ΔG^diss kcal/M	No. of HB and HBE kJ/mol	No. of SB	No. of HI within 5 Å	EE kJ/mol	vWE kJ/mol	TSE kJ/mol	II within 6 Å	*p*-Value
01	30,208.4	2943.3	−16.9	7.6	9/−1.97	1	5	−13.4389	698.76	683.351	1	0.136
02	30,870.4	2275.9	−5.4	−4.9	7/1.6	0	2	150.88	194.884	347.363	0	0.445
03	30,505.3	2650.4	−9.3	−0.3	7/23.69	5	1	−74.352	−80.3236	−130.986	5	0.376
04	31,035.1	2130.5	−11.1	1.4	8/6.3	0	7	−21.5385	285.832	270.594	0	0220
05	30,693.0	2489.1	−13.7	2.1	4/13.13	0	4	22.936	224.77	260.836	0	0.181
06	30,992.7	2166.6	−8.3	−1.5	8/0	0	5	67.633	−19.4197	48.2132	1	0.203
07	30,548.1	2600.7	−4.2	−5.7	8/0.57	0	2	97.1279	85.158	182.856	1	0.435
08	30,681.9	2472.5	−11.2	2.6	11/14.68	0	1	−20.1889	−80.6839	−86.1928	0	0.257
09	30,778.8	2387.3	−12.3	0.7	4/1.58	0	5	−11.9452	−152.368	−162.733	0	0.221
10	30,850.6	2317.6	−7.9	−0.2	11/0.69	2	6	−42.5739	−95.67	−137.554	0	0.425

ΔG^{int}: solvation Free energy gain upon formation of the assembly; ΔG^{diss}: Free energy of assembly dissociation; HB: Hydrogen bonds across the interface; HBE: Hydrogen Bond Energy; SB: Salt bridges across the interface; HI: Hydrophobic Interactions; EE: Electrostatic Energy; vWE: van der Waals Energy; TSE: Total Stabilizing Energy; II: Ionic Interactions.

Table S7. Molecular docking of PR protein to β 1, 3- glucan.

Sl. No.	Binding Energy kcal/mol	Docking Energy kcal/mol	Ligand Efficiency	Inhibition Constant	Inter Molecular Energy kcal/mol	Internal Energy kcal/mol	Hydrogen Bond Formed
1	−3.14	−6.65	−0.09	0.0	−5.32	−1.32	6
2	−3.53	−6.11	−0.1	0.0	−5.71	−0.4	5
3	−1.65	−5.18	0.05	0.06	−3.83	−1.35	3
4	−3.32	−5.65	−0.1	0.0	−5.49	−0.16	5
5	−2.89	−5.78	−0.09	0.01	−5.07	−0.71	1
6	−2.94	−5.43	−0.09	0.01	−5.12	−0.31	4
7	−2.01	−5.6	−0.06	0.03	−4.19	−1.4	5
8	−3.95	−7.08	−0.12	0.0	−6.13	−0.95	5
9	−7.55	−10.47	−0.22	2.94×10^{-6}	−9.73	−0.75	7
10	−5.16	−8.12	−0.15	0.000166	−7.34	−0.79	5

Table S8. Molecular docking of PMSRc protein to β 1, 3- glucan.

Sl. No.	Binding Energy kcal/mol	Docking Energy kcal/mol	Ligand Efficiency	Inhibition Constant	Inter Molecular Energy kcal/mol	Internal Energy kcal/mol	Hydrogen Bond Formed
1	−6.49	−10.22	−0.19	1.76×10^{-005}	−8.67	−1.55	4
2	−4.45	−7.79	−0.13	0.000543	−6.63	−1.15	4
3	−4.18	−7.04	−0.12	0.000858	−6.36	−0.68	4
4	−5.74	−9.18	−0.17	6.22×10^{-005}	−7.92	−1.26	7
5	−6.11	−9.08	−0.18	3.3e−005	−8.29	−0.79	4
6	−5.24	−8.34	−0.15	0.000145	−7.42	−0.93	6
7	−4.73	−8.15	−0.14	0.000343	−6.91	−1.24	7
8	−6.0	−9.29	−0.18	4.03×10^{-005}	−8.17	−1.11	6
9	−6.48	−8.2	−0.19	1.79×10^{-005}	−8.65	−0.45	9
10	−3.89	−6.74	−0.11	0.0	−6.07	−0.67	6

Table S9. Molecular docking of PBPssh2p protein with β 1, 3- glucan.

Sl. No.	Binding Energy kcal/mol	Docking Energy kcal/mol	Ligand Efficiency	Inhibition Constant	Inter Molecular Energy kcal/mol	Internal Energy kcal/mol	Hydrogen Bond Formed
1	−6.01	−8.64	−0.18	3.96×10^{-005}	−8.18	−0.45	3
2	−5.17	−8.82	−0.15	0.000161	−7.35	−1.47	6
3	−5.2	−8.49	−0.15	0.000154	−7.38	−1.11	4
4	−5.02	−7.15	−0.15	0.000207	−7.2	0.05	6
5	−8.03	−11.33	−0.24	1.3×10^{-006}	−10.21	−1.12	5
6	−3.37	−6.19	−0.1	0.0	−5.55	−0.64	3
7	−4.32	−7.63	−0.13	0.000677	−6.5	−1.12	5
8	−4.51	−6.85	−0.13	0.000494	−6.69	−0.16	4
9	−6.23	−9.19	−0.18	2.72×10^{-005}	−8.41	−0.78	5
10	−2.6	−6.25	−0.08	0.01	−4.78	−1.47	5

Table S10. Molecular docking of IAA protein to β 1, 3- glucan.

Sl. No.	Binding Energy kcal/mol	Docking Energy kcal/mol	Ligand Efficiency	Inhibition Constant	Inter Molecular Energy kcal/mol	Internal Energy kcal/mol	Hydrogen Bond Formed
1	−8.92	−11.76	−0.26	2.91×10^{-007}	−11.1	−0.66	8
2	−3.64	−7.11	−0.11	0.0	−5.82	−1.29	5
3	−7.49	−9.34	0.22	3.24×10^{-006}	−9.67	0.33	6
4	−7.3	−10.98	−0.21	4.45×10^{-006}	−9.48	−1.5	2
5	−6.38	−9.83	−0.19	2.11×10^{-005}	−8.56	−1.27	5
6	−4.77	−7.84	−0.14	0.000318	−6.95	−0.89	4
7	−7.4	−10.09	−0.22	3.75×10^{-006}	−9.58	−0.51	6
8	−5.78	−8.72	−0.17	5.76e−005	−7.96	−0.76	3
9	−5.37	−8.07	−0.16	0.000115	−7.55	−0.52	6
10	−7.56	−9.61	−0.22	2.85×10^{-006}	−9.74	0.13	7

Table S11. Molecular docking of PR protein with chitin molecule.

Sl. No.	Binding Energy kcal/mol	Docking Energy kcal/mol	Ligand Efficiency	Inhibition Constant	Inter Molecular Energy kcal/mol	Internal Energy kcal/mol	Hydrogen Bond Formed
1	2.95	−5.12	0.05	-	−2.65	−2.47	2
2	−0.93	−7.46	−0.02	0.21	−6.54	−0.92	2
3	1.34	−5.28	0.02	-	−4.26	−1.02	0
4	1.03	−6.24	0.02	-	−4.57	−1.67	1
5	4.54	−3.29	0.08	-	−1.06	−2.23	1
6	1.44	−3.62	0.03	-	−4.16	0.54	0
7	1.9	−5.57	0.03	-	−3.71	−1.86	3
8	2.94	−3.51	0.05	-	−2.66	−0.86	5
9	1.42	−5.31	0.02	-	−4.18	−1.13	1
10	2.5	−5.46	0.04	-	−3.1	−2.36	2

Table S12. Molecular docking of PMSRc protein with chitin molecule.

Sl. No.	Binding Energy kcal/mol	Docking Energy kcal/mol	Ligand Efficiency	Inhibition Constant	Inter Molecular Energy kcal/mol	Internal Energy kcal/mol	Hydrogen Bond Formed
1	1.15	−6.79	0.02	-	−4.45	−2.33	1
2	1.46	−7.14	0.03	-	−4.14	−3.0	2
3	−1.45	−9.47	−0.03	0.09	−7.06	−2.42	2
4	1.24	−5.52	0.02	-	−4.37	−1.15	4
5	0.62	−7.23	0.01	-	−4.99	−2.24	2
6	−2.06	−7.98	0.04	0.03	−7.66	−0.32	4
7	0.22	−7.46	0.0	-	−5.38	−2.08	4
8	0.52	−7.45	0.01	-	−5.09	−2.36	3
9	−1.92	−7.89	−0.03	0.04	−7.52	−0.37	4
10	−1.49	−8.1	−0.03	0.08	−7.09	−1.0	3

Table S13. Molecular docking of PBPssh2p protein with chitin molecule.

Sl. No.	Binding Energy kcal/mol	Docking Energy kcal/mol	Ligand Efficiency	Inhibition Constant	Inter Molecular Energy kcal/mol	Internal Energy kcal/mol	Hydrogen Bond Formed
1	−1.04	−8.68	−0.02	0.17	−6.65	−2.03	3
2	−0.23	−8.55	0.0	0.67	−5.84	−2.71	2
3	1.36	−6.73	0.02	-	−4.25	−2.48	1
4	1.58	−6.01	0.03	-	−4.02	−1.98	2
5	0.73	−7.44	0.01	-	−4.88	−2.56	4
6	0.9	−6.21	0.02	-	−4.7	−1.51	4
7	−0.63	−7.14	−0.01	0.34	−6.24	−0.91	3
8	−1.37	−8.82	−0.02	0.1	−6.98	−1.85	3
9	−2.29	−9.9	−0.04	0.02	−7.89	−2.01	4
10	1.08	−7.44	0.02	-	−4.53	−2.92	2

Table S14. Molecular docking of IAA protein with chitin molecule.

Sl. No.	Binding Energy kcal/mol	Docking Energy kcal/mol	Ligand Efficiency	Inhibition Constant	Inter Molecular Energy kcal/mol	Internal Energy kcal/mol	Hydrogen Bond Formed
1	0.6	−6.7	0.01	0.0	5.0	−1.7	2
2	0.44	−7.03	0.01	-	5.16	−1.87	2
3	−0.95	−6.14	−0.02	0.2	−6.56	0.42	3
4	0.88	−5.87	0.02	-	−4.73	−1.15	3
5	1.02	−6.54	0.02	-	−4.58	−1.96	4
6	−1.34	−8.3	−0.02	-	−6.94	5.6	2
7	−2.67	−9.52	−0.05	0.01	−8.27	−1.25	4
8	−1.44	−7.84	−0.03	0.09	−7.05	−0.79	1
9	−3.82	−10.64	−0.07	0.0	−9.42	−1.21	2
10	−1.93	−8.84	−0.03	0.04	−7.53	−1.3	3

Table S15. List of primer sequences used for *qRT-PCR* expression studies.

Sl. No	Gene	PRIMERS	Primer Tm	GC Content	Product Size (bp)
1	Indoleacetic acid-induced-like protein (IAA)	F- ACCTCTCGATTCCCTTCTCTCT<br>R- CTTCTCCTTGCTTCCTTTCTCA	59.9 °C<br>60.4 °C	45.5%<br>50.0%	132
2	Pathogenesis-related protein (PR)	F- CTTCTCACCAGCGTTACCATT<br>R- CGGCTTGGAACTTGAAATGA	60.1 °C<br>61.1 °C	45.5%<br>45.5%	150
3	Polyphosphoinositide binding protein ssh2p (PBPssh2p)	F- TTCCTGGTGAGTTTAGTGCTGA<br>R- CAACTTGCTTAACCCTCTGCTC	59.9 °C<br>60.1 °C	50.0%<br>45.5%	159
4	Peptide methionine sulfoxide reductase chloroplastic-like (PMSRc)	F- GAAACTTATCGCACCAGAAAGG<br>R- ATGAGGCAGGATTGACATTAGC	60.1 °C<br>60.5 °C	45.5%<br>45.5%	170
5	Disease resistance rpp13-like protein 1-like (DRrpp13)	F- ATGAAGCCAGCCATCTATCTC<br>R- TGATACCTCCTCCGTTCAAAGT	59.7 °C<br>60.0 °C	45.5%<br>45.5%	142
6	Tubulin	F- GGCTTGTTTCTCAGGTCATTTC<br>R- GAGAGTTGCTCGTGGTAGGC	60.1 °C<br>60.0 °C	45.5%<br>60.0%	175

F: Forward primer; R: Reverse primer.
